# Conditional inactivation of PDCD2 induces p53 activation and cell cycle arrest

**DOI:** 10.1242/bio.20148326

**Published:** 2014-08-22

**Authors:** Celine J. Granier, Wei Wang, Tiffany Tsang, Ruth Steward, Hatem E. Sabaawy, Mantu Bhaumik, Arnold B. Rabson

**Affiliations:** 1 Child Health Institute of New Jersey, Robert Wood Johnson Medical School, Rutgers University, New Brunswick, NJ 08901, USA; 2 Sequencing and Microarray Core Facility, Lewis-Sigler Institute for Integrative Genetics, Princeton University, Princeton, NJ 08854, USA; 3 Waksman Institute and Department of Molecular Biology, Rutgers University, Piscataway, NJ 08854, USA; 4 Rutgers Cancer Institute of New Jersey, Rutgers University, New Brunswick, NJ 08903, USA

**Keywords:** PDCD2, Mouse embryo, Cell cycle, Proliferation, ESCs, p53

## Abstract

PDCD2 (programmed cell death domain 2) is a highly conserved, zinc finger MYND domain-containing protein essential for normal development in the fly, zebrafish and mouse. The molecular functions and cellular activities of PDCD2 remain unclear. In order to better understand the functions of PDCD2 in mammalian development, we have examined PDCD2 activity in mouse blastocyst embryos, as well as in mouse embryonic stem cells (ESCs) and embryonic fibroblasts (MEFs). We have studied mice bearing a targeted PDCD2 locus functioning as a null allele through a splicing gene trap, or as a conditional knockout, by deletion of exon2 containing the MYND domain. Tamoxifen-induced knockout of PDCD2 in MEFs, as well as in ESCs, leads to defects in progression from the G1 to the S phase of cell cycle, associated with increased levels of p53 protein and p53 target genes. G1 prolongation in ESCs was not associated with induction of differentiation. Loss of entry into S phase of the cell cycle and marked induction of nuclear p53 were also observed in PDCD2 knockout blastocysts. These results demonstrate a unique role for PDCD2 in regulating the cell cycle and p53 activation during early embryonic development of the mouse.

## INTRODUCTION

PDCD2 (programmed cell death protein 2) is a highly conserved, zinc finger MYND domain-containing protein of unknown molecular function. PDCD2 RNA was originally identified in thymocytes undergoing programmed cell death (Owens et al., 1991; Vaux and Häcker, 1995), and has been suggested to play a role in apoptotic regulation, particularly in lymphoma cells (Baron et al., 2007; Baron et al., 2010). PDCD2 RNA is widely expressed in normal adult tissues and during embryonic development (Kawakami et al., 1995; Mu et al., 2010; Kramer et al., 2013); however, expression is enriched in mouse neural, embryonic and hematopoietic stem cells (Ramalho-Santos et al., 2002). High levels of expression have also been found in human cancer cells, and persistent expression may be a predictor of clinical relapse in patients with acute leukemia (Barboza et al., 2013). Previous studies have suggested an important role for PDCD2 in development and stem cell activity. Mutations in the *Drosophila melanogaster* PDCD2 homolog, *zfrp8*, result in defective hematopoietic stem cell (HSC) differentiation and altered development of ovarian stem cells (Minakhina et al., 2007; Minakhina and Steward, 2010; Minakhina et al., 2014). Knockdown of PDCD2 in *Danio rerio* results in embryonic lethality and defects in HSC development (Kramer et al., 2013). Consistent with an important role for PDCD2 in stem cells, it was not possible to generate homozygous PDCD2 knockout embryonic stem cells (ESCs) (Mu et al., 2010). PDCD2 knockout in the mouse results in embryonic lethality around 3.5 d*pc* suggesting a critical role in embryonic development at the times of zygotic gene activation and implantation (Mu et al., 2010).

The mechanisms by which PDCD2 exerts its function during mammalian early embryonic development are not known. However, it has been shown that PDCD2 physically interacts with HCF-1 (Host cell factor 1), an important factor in G1 to S transition, suggesting a possible function in cell cycle regulation (Scarr and Sharp, 2002; Tyagi et al., 2007). We generated an inducible knockout of PDCD2 to characterize the effects of PDCD2 deletion on cellular viability and cell cycle. Our results demonstrate an important function of PDCD2 in proliferation, particularly for the G1/S phase transition of the cell cycle in mouse embryonic fibroblasts (MEFs) and ESCs, as well as in mouse blastocyst embryos. Furthermore, PDCD2 loss is accompanied by p53 activation in both blastocyst embryos, and embryo-derived cell lines. Our results demonstrate an important role for PDCD2 in mammalian cell proliferation as early as zygotic gene activation, through regulation of S phase entry, and of p53.

## RESULTS

### A PDCD2 gene trap knockout induces early embryonic lethality

PDCD2-targeted ESCs were purchased from the European Union Conditional Mouse Mutagenesis (EUCOMM) program in order to generate knockout mice. Based on a gene trap strategy (Pettitt et al., 2009), the targeted allele harbors a β-galactosidase cassette, flanked by the Engrailed-2 splicing acceptor and the SV40 polyA site, in the first intron of PDCD2 (supplementary material Fig. S1; “knockout-first” (Skarnes et al., 2011)). This allele, containing a strong splice acceptor, functions as a “gene trap” allele with premature splicing and transcript termination. For simplicity, we refer to this allele as *Pdcd2^−^* (EUCOMM name: *Pdcd2^tm1a(EUCOMM)Wtsi^*). It has been previously shown that PDCD2 knockout in the mouse is embryonic lethal at 3.5 d*pc* (Mu et al., 2010). In order to validate that the “knockout-first” strategy exhibits a functional knockout phenotype, embryonic lethality of homozygous mutants was confirmed (supplementary material Table S1; Fig. S2). These results show that the gene trap mouse line exhibits the early embryonic defects in growth and loss of viability previously described (Mu et al., 2010).

### Generation of conditional (*Pdcd2^flox^*) and “clean” (*Pdcd2^lacZ^*) Pdcd2 knockout alleles

The EUCOMM “knockout-first” allele has been designed for possible use as a conditional knockout allele (supplementary material Fig. S1). This allele harbors two FRT sites flanking the splicing acceptor cassette. By crossing the original knockout line (*Pdcd2^+/−^*) with the *ActB-Flpe* (stock number 003800, from Jackson Laboratory) transgenic line that expresses the Flippase ubiquitously, including in the germ line (Rodríguez et al., 2000), we generated a conditional knockout line harboring *loxP* sites flanking exon 2 of the PDCD2 locus. For simplicity, we refer to this allele as *Pdcd2^flox^* (EUCOMM name: *Pdcd2^tm1c^*). We also crossed male *Pdcd2^+/−^* mice with female *Sox2-Cre* mice (stock number 004783 from Jackson Laboratory), in which Cre is expressed in the germline (Hayashi et al., 2003). This strategy allowed generation of a clean knockout allele following recombination between the 5′ and 3′ *loxp* sites. For simplicity, we refer to this allele as *Pdcd2^lacZ^* (EUCOMM name: *Pdcd2^tm1b^*). We bred these two alleles together to create *Pdcd2^flox/lacZ^*, which will generate a conditional genotype that contains the clean knockout allele and only one Cre targeted allele, minimizing any unintended mosaicism induced upon Cre-mediated recombination.

### Deletion of PDCD2 exon2 in the zygote recapitulates the effects of the PDCD2 knockout mouse

Exon2 of PDCD2 harbors the conserved MYND domain, a hypothetical functional domain of PDCD2. In order to test whether this domain is critical for PDCD2 function, we induced the conditional knockout of *Pdcd2* exon 2 in the germline. *Pdcd2^+/flox^* males were crossed with *Sox2-Cre* females. The allele generated by recombination of the conditional allele was designated *Pdcd2^Δexon2^* (EUCOMM name: *Pdcd2^tm1d^*) (supplementary material Fig. S1). Embryonic lethality of homozygous mutants *Pdcd2^Δexon2/Δexon2^* was observed, similarly as observed with *Pdcd2^−/−^*, at peri-implantation stage (supplementary material Table S2). These results provide validation for the use of the exon2 deletion as a strategy to conditionally knockout PDCD2 under temporally defined or cell-type specific conditions.

### Loss of PDCD2 in mouse embryonic fibroblasts results in defects in entry into the S phase of the cell cycle

In order to better understand the cellular defects induced by loss of PDCD2, we generated *in vitro*, inducible knockout MEFs. *Pdcd2^+/flox^; R26-Cre-ER^T2^* (Ventura et al., 2007) and *Pdcd2^+/lacZ^* mice were intercrossed in order to generate Tamoxifen (Tam)-inducible knockout MEFs. PDCD2 protein and RNA levels were analyzed every 24 h (supplementary material Fig. S3A,B). PDCD2 RNAs containing exon 2 show a dramatic decrease in the *Pdcd2^flox/lacZ^* MEFs, starting at 24 h, with no detectable expression at 48 h post Tam (supplementary material Fig. S3A); instead, a shorter RNA was observed using PCR primers from exons 1–3, corresponding to exon 2-deleted RNA. Following Tam treatment of *Pdcd2^flox/lacZ^* MEFs, PDCD2 protein is undetectable after 48 h (supplementary material Fig. S3B; Western blot upper panel; quantitation of PDCD2 band intensity lower panel). Although we expected that a truncated PDCD2 protein might have been detected (due to exon2 deletion), we did not observe any smaller PDCD2 species in these cells. This result suggests that loss of exon 2 of PDCD2 may result in loss of PDCD2 protein synthesis/stability.

**Fig. 1.**
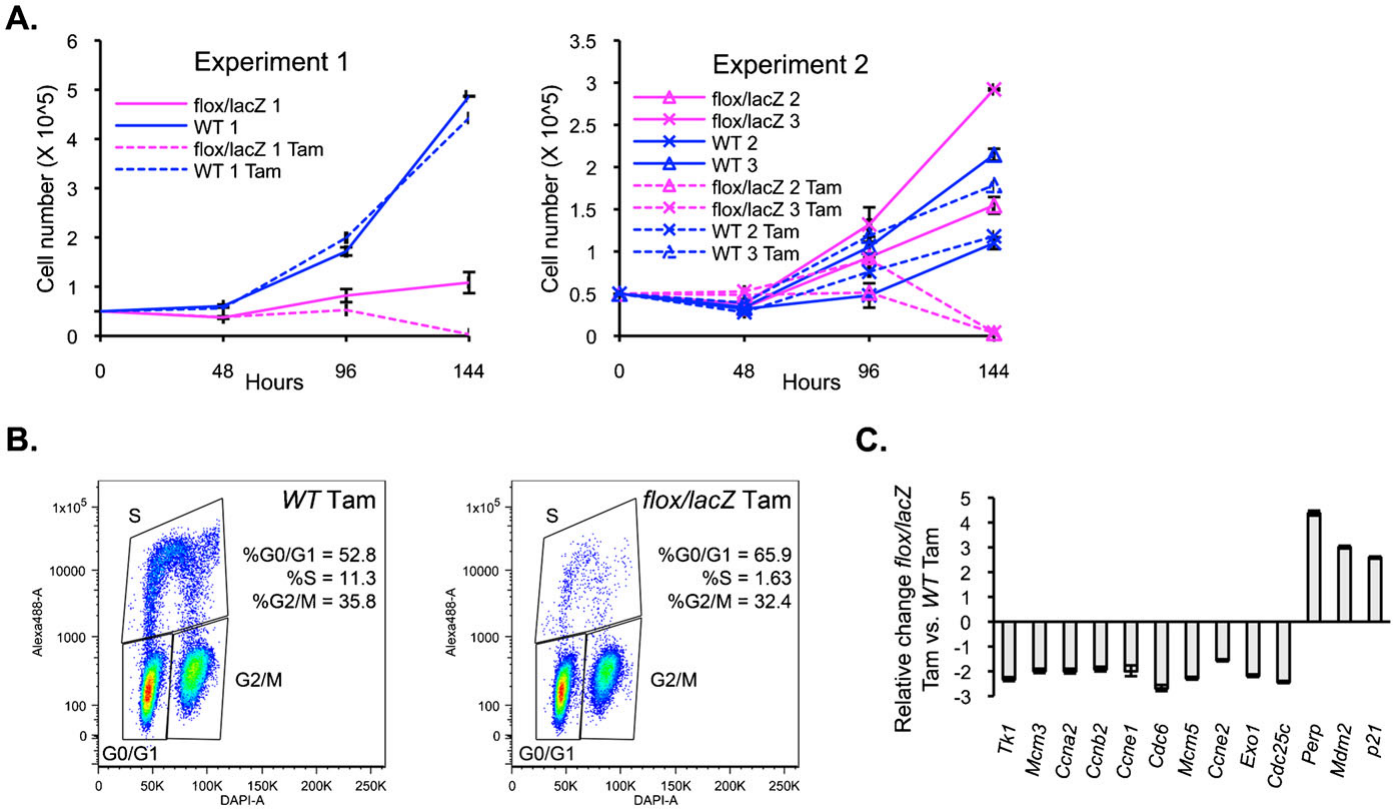
Inducible knockout of PDCD2 in MEFs results in loss of cell growth and defects in transition from G1 to S phase of the cell cycle. (A) Growth curve of inducible knockout and control MEFs, treated or untreated with Tamoxifen (Tam). Two independent experiments, Experiment 1 (left panel) and Experiment 2 (right panel), were performed using three WT and three *Pdcd2^flox/lacZ^* MEF cell lines in total. In Experiment 1, *Pdcd2^flox/lacZ^* MEFs (line 1) exhibited slowed growth compared to *WT* MEFs, in the absence of Tam. In Experiment 2, two *Pdcd2^flox/lacZ^* MEFs (lines 2, 3) showed similar growth to that of two other WT MEF lines. In both experiments, Tam-treated *Pdcd2^flox/lacZ^* MEFs exhibit a cessation of growth following Tam treatment. (B) Flow cytometry analysis of cell proliferation after 1 h of EdU incorporation. Data are presented as plots for DAPI versus EdU staining (alexa-488). EdU positive cells (i.e. S phase cells) comprised 11.3% of total *Pdcd2^+/+^* cells after doublet exclusion as compared with only 1.63% of *Pdcd2^flox/lacZ^* cells, 96 h after Tam treatment. (C) qPCR analysis of E2F and p53 target gene RNA levels. Results represent the fold change of *Pdcd2^flox/lacZ^* compared to *Pdcd2^+/+^* cells 72 h after Tam treatment. Experiments represented by panels B and C were performed using WT and KO lines 3.

Having validated the loss of PDCD2 protein expression in the *Pdcd2^flox/lacZ^; R26-Cre-ER^T2^* MEFs, we analyzed the growth of these cells by plating mutant (*Pdcd2^flox/lacZ^* line) as well as WT control MEFs at low density, in presence or absence of Tam for 24 h, and counting cells every 24 h (Fig. 1A). We performed two independent growth analyses comparing the growth of three different WT and *Pdcd2^flox/lacZ^* MEFs. WT line1 MEFs grew normally, in the presence or absence of Tam. In the absence of Tam, *Pdcd2^flox/lacZ^* line 1 MEFs showed a significant decrease in growth rate compared to the WT controls, suggesting a possible dose effect of the loss of one allele of *Pdcd2* in these cells (Fig. 1A, left panel). In a second independent experiment, no evidence of reduced growth was seen in two additional *Pdcd2^flox/lacZ^; R26-Cre-ER^T2^* MEF lines (Fig. 1A, right, *Pdcd2^flox/lacZ^* lines 2, 3), arguing against a role for haploinsufficiency in PDCD2 knockout mice. Consistently, following Tam treatment, all three *Pdcd2^flox/lacZ^* MEFs showed a loss of growth and a decline in cell number between 96 and 120 h after treatment. Compared to control cells, the knockout MEFs exhibited an irregular, elongated morphology, with loss of adherence (supplementary material Fig. S3C), and failed to reach confluence. No obvious floating or dead cells were observed.

In order to determine if the growth arrest in the knockout MEFs was associated with cell cycle defects, DNA content as well as incorporation of EdU was analyzed. EdU incorporation, a marker of new DNA synthesis, was dramatically decreased in *Pdcd2^flox/lacZ^* MEFs 96 h after Tam treatment as compared to *WT* cells (i.e. 1.6% S phase in *Pdcd2^flox/lacZ^* cells compared to 11.6% in *WT* cells), and was accompanied by an increase in G1 phase cells (Fig. 1B).

Inducible knockout primary MEFs (lines 1) were immortalized by repeated re-plating (Xu, 2005). *Pdcd2^flox/lacZ^; R26-Cre-ER^T2^*, as well as control immortalized MEFs were plated at low density, in presence or absence of Tam for 24 h, and cell numbers analyzed over time (supplementary material Fig. S4). Immortalized PDCD2-inducible knockout MEFs show the same growth pattern as the primary knockout MEFs, with dramatic loss of growth following Tam treatment, confirming the role of PDCD2 in proliferation of embryonic fibroblasts.

### RNA-seq on PDCD2 knockout MEFs reveals defects in cell cycle progression and p53 induction

In order to establish a link between the defects in cellular proliferation associated with PDCD2 loss, and changes in cellular gene expression, an RNA-seq analysis (next generation sequencing of RNAs) was performed (supplementary material Fig. S5; Tables S3, S4, S5, S6). Total RNA from WT and *Pdcd2^flox/lacZ^* MEFs was isolated 72 h after Tam treatment. For each genotype, RNA-seq reactions were performed using ribosomal RNA-depleted (labeled as “Ribo^−^” or “r”) RNA samples, and separately, using polyA-enriched (“polyA^+^” or “a”) samples. As a validation of the RNA-seq, we compared the representation of RNA sequencing reads from different PDCD2 exons in the Tam-treated, WT and *Pdcd2^flox/lacZ^* MEFs (supplementary material Fig. S5A). As expected, the *Pdcd2^flox/lacZ^* MEFs showed a marked loss of sequence reads corresponding to *Pdcd2* exon 2. Genes that changed >1.5-fold following Tam treatment of *Pdcd2^flox/lacZ^* MEFs as compared with wild type are shown in supplementary material Table S3 (polyA^+^), Table S4 (Ribo^−^), and Table S5 (changed in both polyA^+^ and Ribo^−^). Hierarchical clustering of the genes in supplementary material Table S5 (supplementary material Fig. S5B) shows clustering of gene expression within each cell type (i.e. WT versus *Pdcd2^flox/lacZ^*).

Gene function analyses were performed on the 215 genes that exhibited 2.0-fold or greater changes in gene expression in both the polyA^+^ and Ribo^−^ preparations. Pathway analysis [DAVID software, http://david.abcc.ncifcrf.gov/home.jsp (Huang da et al., 2009a; Huang da et al., 2009b)], using the KEGG pathway database showed a strongly statistically significant enrichment of genes associated with cell cycle (p = 8.1E−19) among the 165 genes whose expression decreased following deletion of PDCD2 (Table 1). At least 64 of the downregulated genes were previously identified in the literature as E2F target genes (supplementary material Table S6). The Ribo^−^ sets of RNA-seq data (including non-polyadenylated RNAs) showed a pronounced downregulation (more than 2-fold) of the replication-dependent, histone locus genes on mouse chromosome 1, also known to be E2F targets (supplementary material Table S4). These results are all consistent with a major effect of PDCD2 loss in MEFs resulting in inhibition of entry into the S phase of the cell cycle.

**Table 1.**
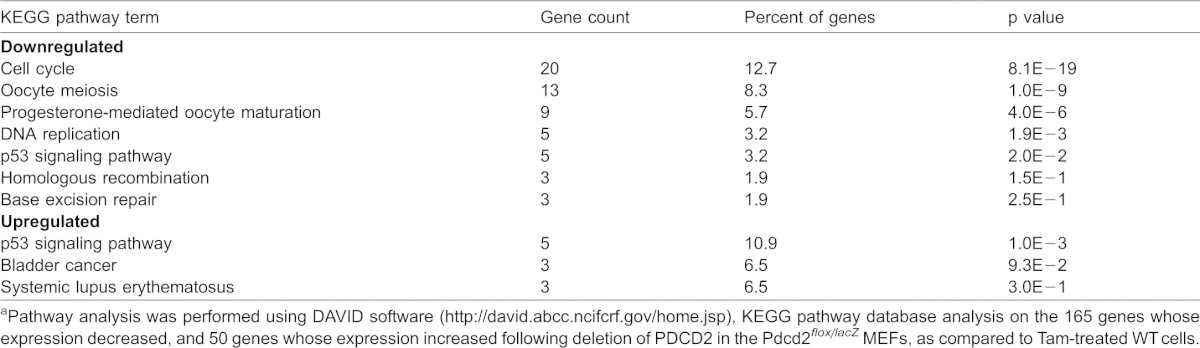
Pathway analysis of altered gene expression associated with PDCD2 knockout^a^

In order to confirm the RNA-seq results, quantitative PCR (qPCR) was performed on total RNA samples from *Pdcd2^flox/lacZ^* MEFs, as compared with wild-type MEFs at 72 hrs following Tam (Fig. 1C). Ten different known E2F target genes (Helin, 1998; Bracken et al., 2004; Conboy et al., 2007; Xu et al., 2007) all showed between 1.5–3.0-fold decreases in gene expression associated with the loss of PDCD2, similar to the RNA-seq data for these genes.

DAVID analysis using the KEGG pathway database of the 50 genes whose expression increased 2.0-fold or more following PDCD2 knockout (Table 1), showed a significant enrichment of genes in the p53 pathway (5 genes, p value  =  3.1E−05; the only pathway with a p value <E−03). These included the p53 autoregulator MDM2, the pro-apoptotic PERP gene, and the cdkn1a/p21 cell cycle inhibitor. qPCR on total RNA samples from *Pdcd2^flox/lacZ^* MEFs at 72 hrs following Tam treatment showed a 2.5–4.5-fold induction of Mdm2, Perp and p21, consistent with the RNA-seq results (Fig. 1C). Increased p53 protein expression was also observed by Western blot analysis (supplementary material Fig. S3D) and immunofluorescence showed significantly more nuclear p53 in knockout MEFs compared to WT, with up to 80% of KO cells having nuclear p53 (supplementary material Fig. S3E). Interestingly, as shown in Table 1, p53 pathway genes were also enriched among the genes whose expression decreased following PDCD2 knockout. Importantly, while the genes that increased were direct p53 targets (such as MDM2, PERP and p21), genes that decreased were not direct p53 targets, but instead were downstream regulators of the cell cycle, including cyclin E2, cyclin B2, and cdc2. Thus, the effects of PDCD2 loss appear to involve induction of p53 and the p53 direct target gene, p21, with reduction of downstream, cell cycle promoting genes.

### Loss of PDCD2 in mouse embryonic stem cells results in loss of proliferation and failure of S phase entry

**Fig. 2.**
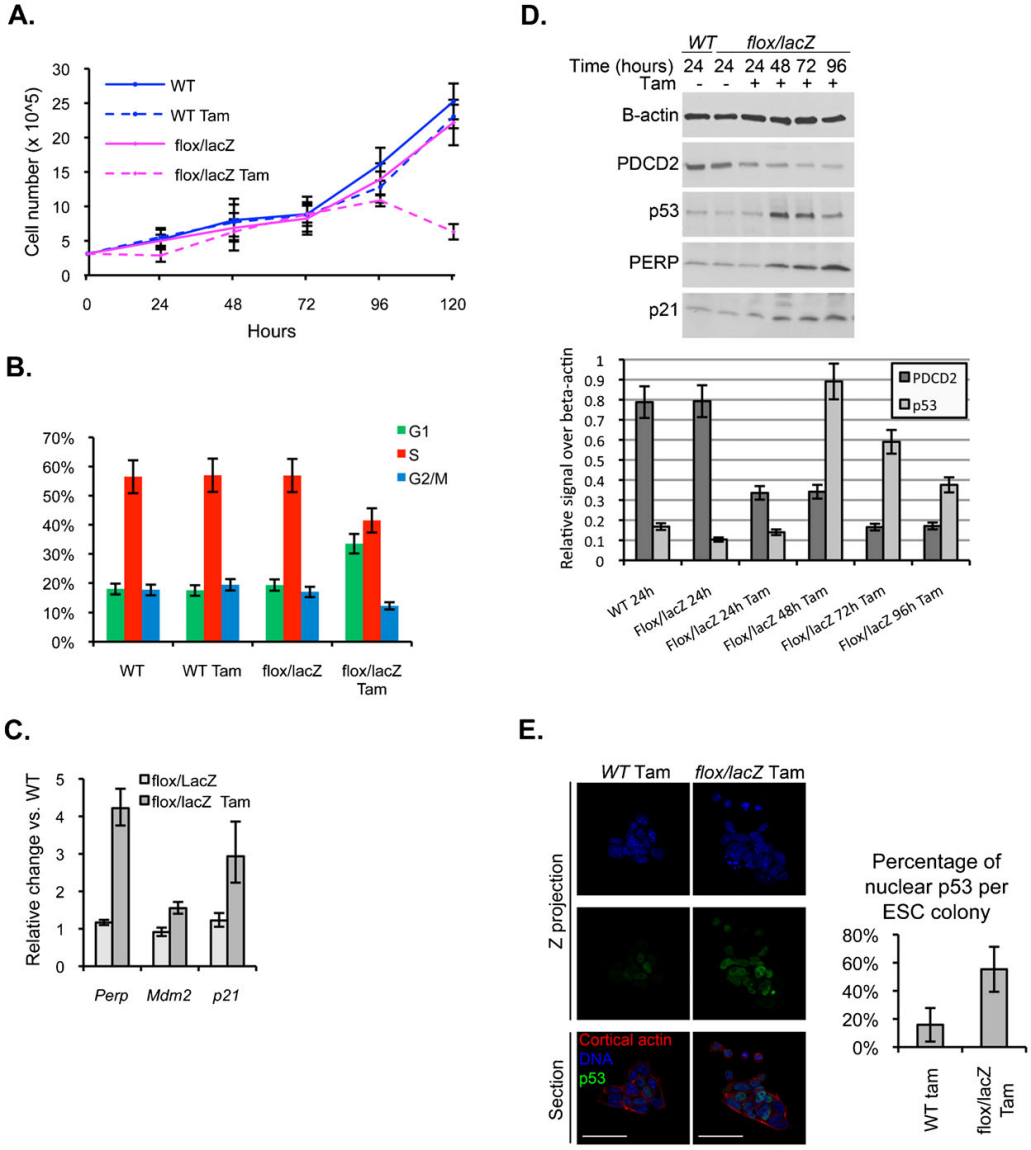
*Ex vivo* inducible knockout of PDCD2 in ESCs results in loss of S phase entry and increased p53. (A) Growth curve of inducible knockout and WT ESCs in presence or absence of Tamoxifen. Tam-treated *Pdcd2^flox/lacZ^* ESCs exhibit a cessation of growth 96–120 h following Tam treatment. (B) Cell cycle analysis of *Pdcd2^+/+^* and *Pdcd2^flox/lacZ^* ESCs after 120 h after Tam treatment. Tam treatment of *Pdcd2^flox/lacZ^* ESCs reduced percentages of cells in S phase and G2/M, with increased numbers of cells in G1. Histograms summarize raw cell cycle data, shown in supplementary material Fig. S6A. (C) qPCR analysis of p53 target gene RNA levels. Results represent the fold change of *Pdcd2^flox/lacZ^* (with and without Tam) compared to *Pdcd2^+/+^* cells (+Tam) 96 h after Tam treatment. (D) Western blot analysis of PDCD2, p53, PERP and p21 protein levels in *Pdcd2^flox/lacZ^* cells 24 to 96 h after Tam treatment. β-actin protein levels are shown as a loading control. Additional controls are shown in supplementary material Fig. S6C. Bottom panel shows quantification of PDCD2 and p53 protein level normalized to β-actin levels. (E) Immunofluorescence for p53 protein 72 h after Tam treatment of WT and *Pdcd2^flox/lacZ^* ESCs. Left panel: confocal acquisitions of ESCs stained for DNA (Hoechst, blue), cortical actin (Phalloidin, red) and p53 (green); right panel: quantification of percentage of cells with nuclear p53 cells per ESC colony (TCN = total cell number, quantified based on Hoechst staining). Significantly more cells with nuclear p53 are present in *Pdcd2^flox/lacZ^* ESCs (average 55.35%, N = 5 colonies) than WT ESCs (average 16.02%, N = 6 colonies), (p = 0.001, *t* test) 72 h after Tam treatment. Scale bars: 50 µm.

Since PDCD2 knockout is embryonic lethal at the blastocyst stage, and PDCD2 has been proposed to be stem cell-associated gene product, we were interested in examining the functions of PDCD2 in ESCs. It was previously shown that it was not possible to generate homozygous PDCD2 knockout ESCs, yet siRNA-mediated knockdown (with a small amount of residual PDCD2 expression) had little effect on ESC growth (Mu et al., 2010). Therefore, we generated inducible knockout ESCs following the same strategy as employed for inducible knockout MEFs. *Pdcd2^+/flox^; R26-Cre-ER^T2^* and *Pdcd2^+/lacZ^* mice were intercrossed in order to generate Tam-inducible knockout ESCs, isolated from 3.5 d*pc* embryos. *Pdcd2^flox/lacZ^; R26-Cre-ER^T2^*, as well as WT ESCs, were plated at low density, in presence or absence of Tam for 24 h, and cells were counted every 24 h (Fig. 2A). WT ESCs grew normally, independent of the presence of Tam. Interestingly, in contrast to the MEFs, *Pdcd2^flox/lacZ^* ESCs, in absence of Tam displayed a growth rate comparable to the WT controls, showing no dose effect of the loss of one allele of PDCD2 in these cells. In presence of Tam, *Pdcd2^flox/lacZ^* ESC cell number abruptly declined between 96 and 120 h after treatment.

The cell cycle of ESCs is composed of a long S phase and a shortened G1 phase (Savatier et al., 1994; Savatier and Afanassieff, 2002; Neganova and Lako, 2008). As expected, the control ESCs showed 50 to 60% of cells in S phase (Fig. 2B; supplementary material Fig. S6A). However, similar to the inducible knockout MEFs, cell cycle analysis showed increased G1, and decreased S and G2/M in *Pdcd2^flox/lacZ^* ESCs treated with Tam, compared to untreated controls and WT (Fig. 2B; supplementary material Fig. S6A). Molecularly, ESCs have been reported to exhibit constitutive activation of CDK2/Cyclin E with constitutive Rb hyperphosphorylation that is not dependent upon CDK/Cyclin D activity (Savatier et al., 1996; Savatier and Afanassieff, 2002; Neganova and Lako, 2008). In view of these differences, we examined the expression of a subset of the E2F target genes that were reduced in the PDCD2-deficient MEFs. These E2F target genes generally exhibited less than a 2-fold decrease in the ESCs following PDCD2 deletion, except for Ccne2, which exhibited a 3-fold decrease (supplementary material Fig. S6B). Thus, despite the fundamental differences between ESC and MEF cell cycles, PDCD2 knockout in ESCs also leads to failure in S phase entry, associated with a slight decrease in the expression of some E2F target genes.

### PDCD2 loss in ESCs is associated with increased p53 protein

Interestingly, similar to the PDCD2-deficient MEFs, expression of RNAs for the p53 targets p21, Mdm2 and PERP was increased in PDCD2-deficient ESCs (Fig. 2C). We therefore determined the levels of p53 protein following PDCD2 loss in *Pdcd2^flox/lacZ^* ESCs. We performed Western blot analyses for p53 protein levels, as well as for selected p53 target genes. Within 48 hours of Tam treatment (at a point when PDCD2 levels had decreased 60%), markedly increased p53 protein levels (>9-fold) were already observed (Fig. 2D, upper-Western Blot, below-quantitation; supplementary material Fig. S6C). p53 levels remained elevated to 96 hours post-Tam. Although relatively little is known about p53 function in ESCs, nuclear translocation of cytoplasmic p53 has been associated with p53 activation in these cells (Solozobova et al., 2009). We therefore examined levels of nuclear p53 in *Pdcd2^flox/lacZ^* ESCs following Tam (Fig. 2E,F). Increased numbers of PDCD2-deficient ESCs exhibited nuclear p53 following Tam treatment (up to 60% of cells), as compared to treated control cells.

Induction of p53 and expression of p53 target genes are generally associated with induction of cell cycle arrest and apoptosis. PDCD2 loss in both ESCs and MEFs leads to induction of p53 target genes known to be associated with each outcome: Cdkn1a/p21 mediates p53-induced cell cycle arrest and PERP is a p53-induced, pro-apoptotic gene. As shown above, DNA content analysis of both MEFs and ESCs depleted of PDCD2 revealed a failure to enter S phase; however, neither MEFs (Fig. 1B) nor ESCs (Fig. 2B; supplementary material Fig. S6A), showed increased cells containing sub-G1 amounts of DNA, suggesting that PDCD2 loss did not lead to apoptosis in these cells.

### Loss of PDCD2 in ESCs does not lead to premature differentiation

**Fig. 3.**
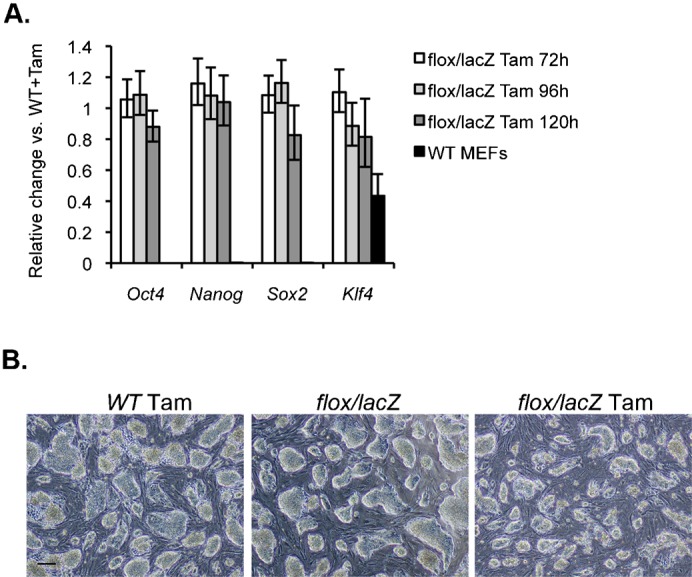
PDCD2 knockout in ESCs does not induce differentiation. (A) qPCR analysis of Oct4, Nanog, Sox2 and Klf4 gene RNA levels. Results represent the fold change of RNA in *Pdcd2^flox/lacZ^* compared to *Pdcd2^+/+^* cells 72 h, 96 h and 120 h after Tam treatment. WT MEFs have been used as negative controls for these markers of pluripotency. (B) Brightfield pictures showing morphology of the ES cells grown for 96 h. Scale bar: 50 µm.

Studies of ESCs have shown that their rapid cell division is associated with an unusual cell cycle composed of a predominant S phase and a markedly shortened G1 phase, compared to differentiated cells (Savatier et al., 1994; Savatier et al., 1996; White et al., 2005). As ESCs differentiate, the length of the G1 phase increases (Savatier et al., 1996; White et al., 2005). PDCD2-depleted ESCs demonstrated a substantial elongation of the G1 phase. Moreover, it has been shown that upon differentiation of ESCs, the p53 target, p21, is upregulated (Sabapathy et al., 1997). We therefore investigated if PDCD2-depleted cells displayed a higher susceptibility to differentiation. If true, the increase in G1 length could be a consequence of differentiation of PDCD2-depleted cells. We examined the expression of the pluripotency markers, Oct4, Sox2, Klf4 and Nanog (Fig. 3A) in *Pdcd2^flox/lacZ^* ESCs versus WT ESCs after 72 h, 96 h and 120 h of Tam treatment. WT MEFs were used as a negative control. The levels of all four pluripotency markers were significantly higher in ESCs than in MEFs (from 2-fold for Klf4 to >10,000-fold for Oct 4). Importantly, no significant changes in their levels were detected in PDCD2-depleted ESCs compared to WT ESCs. The morphology of the ESCs colonies was also monitored (Fig. 3B). PDCD2 knockout colonies appear smaller than the control cells, but do not exhibit morphological features of differentiation. Thus, PDCD2 loss did not itself induce differentiation of ESCs.

### PDCD2 knockout embryos contain reduced numbers of cells in cell cycle S phase and more cells in G1 phase

**Fig. 4.**
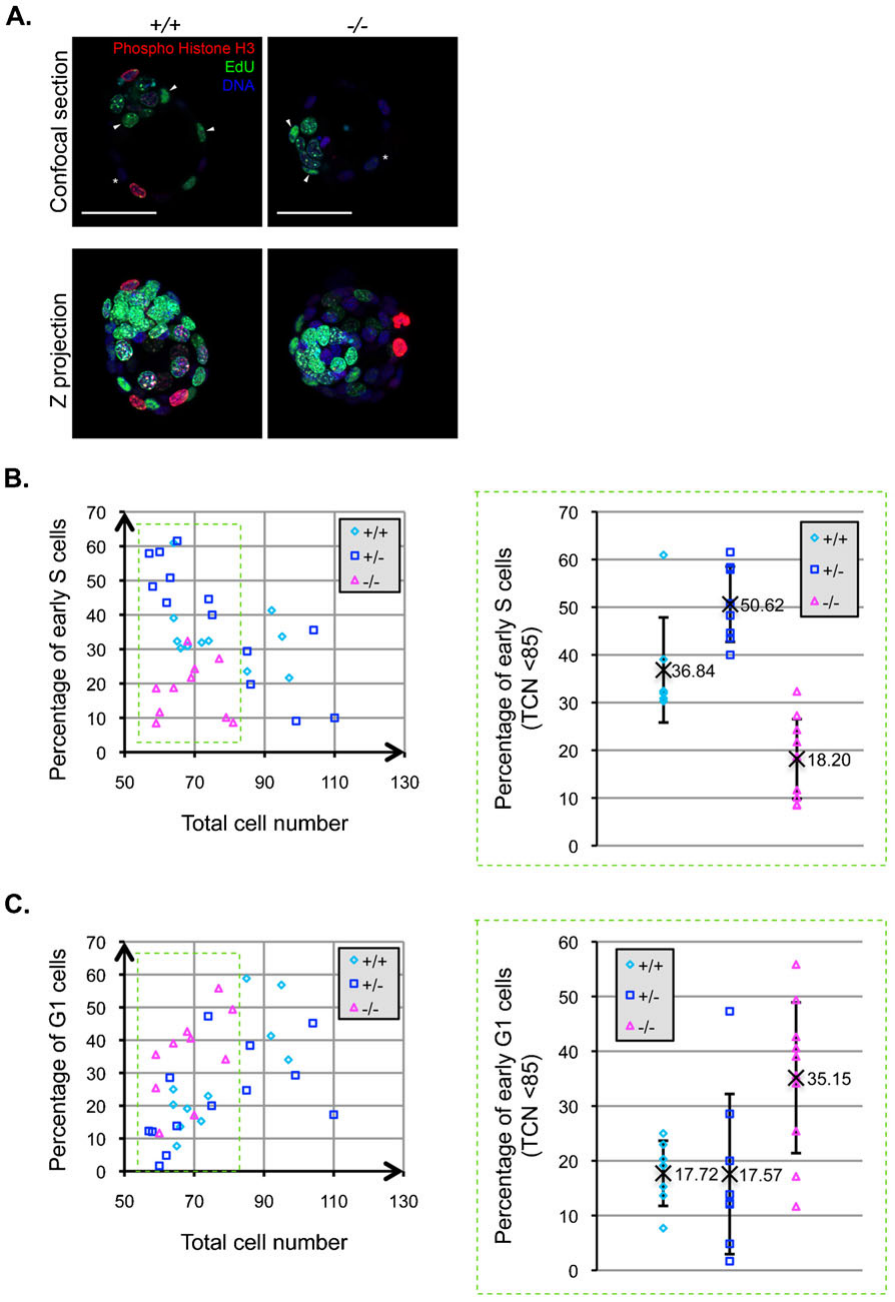
PDCD2 knockout in the embryo results in defects in S phase entry. (A) Confocal images showing EdU incorporation (green), immunofluorescence for Phospho-Histone H3 (red) and Hoechst labelling (blue). Arrowheads indicate a subset of early S phase cells (diffuse EdU positive, Phospho-Histone H3 negative) and asterisks indicate examples of G1 phase cells (EdU negative, Phospho-Histone H3 negative). (B) Left panel represents the percentage of cells in early S phase (diffuse EdU positive, Phospho-Histone H3 negative) relative to the total cell number (TCN-Hoechst positive cells). Right panel represents the percentage of early S phase among embryos that have a TCN of less than 85 (derived from data in the green dashed box of left panel). Homozygous knockout embryos contain significantly fewer S phase cells (average 18%, N = 10) than WT (average 37%, N = 7, p = 0.001 by *t* test) and heterozygous (average 51%, N = 8, p = 0.0004 by *t* test). (C) Left panel represents the percentage of cells in early G1 phase (EdU negative, Phospho-Histone H3 negative) relative to the total cells number (TCN-Hoechst positive cells). Right panel represents the percentage of early G1 phase among embryos that have a TCN of less than 85. Homozygous knockout embryos contain significantly fewer S phase cells (average 35%, N = 10) than WT (average 18%, N = 7, p = 0.006 by t test) and heterozygous (average 18%, N = 8, p = 0.01 by t test). Scale bars: 50 µm.

Inducible knockout of PDCD2 in ESCs resulted in failure of entry into S phase of the cell cycle, and PDCD2 knockout embryos exhibited a significant defect in growth. Furthermore, similar to ESCs, peri-implantation embryo cells exhibit a predominant S phase with a brief G1 phase (Artus and Cohen-Tannoudji, 2008). We decided to analyze the cell cycle phenotype of cells comprising the PDCD2 knockout blastocyst (3.5 d*pc*), using a dual immunofluorescence approach. We stained cells in the blastocyst for the presence of Phospho-Histone H3 (Ser10) and for EdU incorporation, adapting a method based on the Click iT EdU imaging kit (Invitrogen). By BrdU or EdU labeling, it is possible to distinguish between “early”, “mid” and “late” S phase, based on the qualitative distribution of the incorporated label (diffuse staining in early S phase versus the presence of large punctate structures in later S phase) (Dahm and Hübscher, 2002; Quivy et al., 2008; Fu et al., 2013). Moreover, there is a correlation between phosphorylation of Histone-H3 serine 10 (H3Ser10) and cell cycle stage. H3Ser10 phosphorylation is initiated during G2 and maintained up to the telophase of the dividing cell (Hendzel et al., 1997; Prigent and Dimitrov, 2003). In a WT blastocyst, about 50% of the cells are in S phase (Artus et al., 2005). We employed this dual staining approach to quantify the percentage of cells in G1 (negative for EdU, negative for Phospho-Histone H3), and the percentage of cells entering S phase (i.e. “early” S phase exhibiting diffuse EdU staining and negative for Phospho-Histone H3) for individual embryos (Fig. 4A).

In the WT and heterozygous embryos, the percentage of S phase cells decreases in inverse proportion to increasing total cell number, i.e. with the size of the embryos (Fig. 4B, left panel). The opposite result is observed for the number of G1 phase cells; as the embryo grows, the percentage of cells in G1 increases (Fig. 4C, left panel). As shown in supplementary material Fig. S2A, the total cell number of the PDCD2 knockout embryos fails to exceed a certain size (approximately 79 cells in these experiments). Comparison of embryos smaller than 85 total cell nuclei revealed significant differences in cell cycle phase (Fig. 4B,C, right panels). WT and heterozygous embryos contained averages of 37% and 51% of cells in S phase as compared with approximately 18% S phase cells in the PDCD2 knockout embryos (Fig. 4B, right panel). Moreover, PDCD2 knockout embryos contained 35% G1 phase cells, significantly more than the littermate controls that exhibited 18% of cells in G1 (Fig. 4C, right panel). The fact that the number of cells in S phase decreases and the number in G1 increases is unlikely to be due to increased cell death or exit from the cell cycle. Thus, at given embryo sizes, PDCD2 knockout embryos contain fewer S phase cells and more G1 phase cells, than the WT and heterozygous littermate controls.

### PDCD2 knockout embryos exhibit increased nuclear p53

**Fig. 5.**
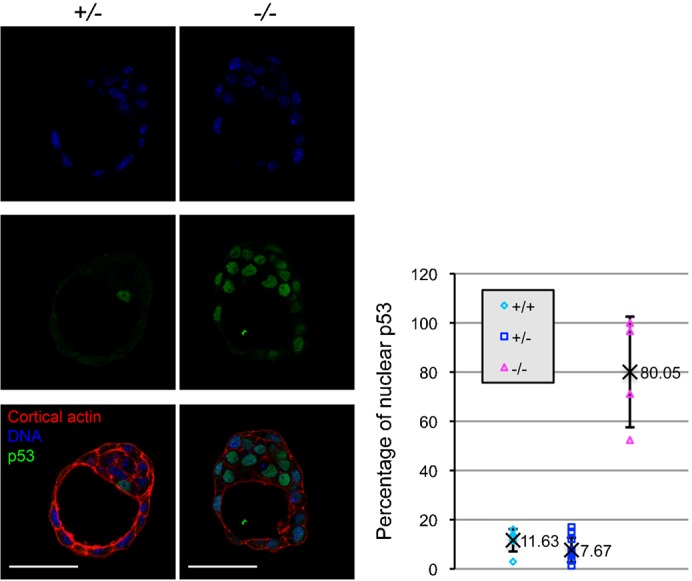
PDCD2 knockout in early embryos induces nuclear p53. Left panel: confocal sections of heterozygous (+/−) and homozygous (−/−) PDCD2 knockout 3.5 d*pc* embryos stained for DNA (Hoechst, blue), cortical actin (Phalloidin, red) and p53 (green); right panel: quantification of percentage nuclear p53 cells per embryo (TCN quantified based on Hoechst staining). Homozygous knockout embryos contain significantly more nuclear p53 cells (average 80%, N = 4) than WT (average 11.6%, N = 11, p = 7.E−5 by *t* test) and heterozygous embryos (average 7.7%, N = 4, p = 9.E−8 by *t* test). Scale bars: 50 µm.

The inducible knockout of PDCD2 resulted in increased p53 protein in MEFs and ESCs, and increased nuclear p53 in ESCs. Since we also observed defective entry into S phase of the cell cycle after knockout of PDCD2 in early mouse embryos, we were interested in determining if nuclear p53 was induced in PDCD2 knockout embryos. 3.5 d*pc* embryos were stained by immunofluorescence for p53 (Fig. 5). An average of 11.6% of nuclei in WT embryos (N = 6) and 7.7% for heterozygous embryos (N = 11) exhibited nuclear p53 staining. In marked contrast, 80% of the nuclei in the homozygous PDCD2 knockout embryos (N = 4) showed strong staining for nuclear p53. Thus, the loss of PDCD2 is associated with strong induction of p53 in early mouse embryos.

## DISCUSSION

Although PDCD2 is highly conserved across evolution [with close to 40% amino acid conservation, between humans and *Drosophila* (Minakhina et al., 2007)], the function of PDCD2 remains mysterious. The initial study of the knockout of PDCD2 in the mouse showed that it is necessary for viability of the blastocyst and for the ability to isolate ESCs (Mu et al., 2010). Our studies have shown that PDCD2 plays a critical role in entry into S phase of the cell cycle in MEFs, ESCs, and in the blastocyst embryo. Loss of PDCD2 was associated with induction of p53 and expression of its target genes. These studies point to a unique essential role of PDCD2 in controlling cell cycle of embryonic cells, likely mediated through the p53 pathway.

Although the expression of PDCD2 is enriched in embryonic, neural and hematopoietic stem cells (Ramalho-Santos et al., 2002), PDCD2 is ubiquitously expressed in different tissues during the course of embryonic development (Kawakami et al., 1995; Mu et al., 2010; Kramer et al., 2013). Our studies have shown that PDCD2 is required for proliferation of MEFs as well as ESCs, thus demonstrating its effects on the proliferation of both pluripotent and more differentiated cells. In previous studies, knockdown of PDCD2 in human lung cancer cells induced slowing of the cell cycle without an obvious specific defect (Barboza et al., 2013). Our results show that PDCD2 knockout in embryonic primary cell models is associated with arrest at the G1/S transition of the cell cycle. The exact mechanism by which PDCD2 knockout inhibits S phase entry still needs to be determined. Others have speculated that the reported interaction of PDCD2 with HCF-1 (Host Cell Factor-1) (Scarr and Sharp, 2002), known to be a key regulator of G1/S transition through its interactions with E2F proteins (Tyagi et al., 2007), may play an important role in blocking cell cycle (Barboza et al., 2013). If so, PDCD2 would not be expected to affect upstream events, such as CDK activation and Rb phosphorylation. Alternatively, the association of PDCD2 loss with induction of p21/CDKN1A, a p53 target that is a key inhibitor of cyclin-CDK2 and cyclin-CDK4 complexes (Bieging and Attardi, 2012), may be sufficient to explain the defect in S phase entry.

We have shown that PDCD2 knockout in ESCs, MEFs and 3.5 d*pc* embryos leads to increased p53 protein levels and p53 nuclear localization. Moreover, in ESCs and MEFs, the p53 target genes, p21, Mdm2 and PERP were upregulated. Activation of p53 often occurs in response to cellular stress (Bieging and Attardi, 2012). PDCD2 knockout may induce a common cellular stress response pathway, in turn leading p53 activation. One of the most important causes of p53 induction is DNA damage (Bieging and Attardi, 2012; Reinhardt and Schumacher, 2012). Recent results suggest that the *Drosophila* PDCD2 homologue Zfrp8 regulates the piRNA pathway in ovarian stem cells (Minakhina et al., 2014); loss of Zfrp8 was associated with increased expression of transposable elements, which can induce DNA damage upon integration. Another cause of p53 induction is stress induced by reduced protein translation, secondary to multiple causes including failure to adequately synthesize ribosomes (Deisenroth and Zhang, 2010). The yeast gene exhibiting the closest homology to PDCD2, Yol022/TSR4, has been implicated in the proper processing of the 20S precursor of 18S ribosomal RNA (Li et al., 2009). Alternatively, it is possible that PDCD2 interacts directly with the p53 signaling pathway. p53 activity is finely regulated post-translationally, by degradation by the proteasome through MDM2-driven ubiquitination (Kruse and Gu, 2009; Lee and Gu, 2010). PDCD2 has been reported to interact with a number of regulators of the ubiquitin pathway, including the ubiquitin specific peptidases USP49 and USP43 (http://string-db.org), and the ubiquitin ligase, Parkin (Fukae et al., 2009). Thus, PDCD2 may play a role in regulation of p53 ubiquitination. Of note is the fact that other MYND domain-containing proteins participate in regulation of ubiquitin regulated pathways (Ju et al., 2008). The fact that knockout of the primary p53 regulator, MDM2, is embryonic lethal at peri-implantation stages, similar to the PDCD2 knockout (Jones et al., 1995; Montes de Oca Luna et al., 1995), might support more direct effects of PDCD2 on the p53-MDM2 pathway.

We have shown by a combination of gene expression studies and morphologic analysis that the cell cycle arrest observed with PDCD2 knockout in ESCs is not a consequence of premature differentiation. It has been shown that as ESCs differentiate, the G1 phase elongates, and the cell cycle becomes regulated by the Rb-E2F pathway (Savatier et al., 1996; White et al., 2005). Our results show G1 phase elongation in ESCs without premature differentiation. In other studies, genetic manipulations that artificially lengthen G1 in ESCs by overexpression of the Cdk inhibitor proteins, p21 or p27, do not induce differentiation (Li et al., 2012). Our results thus confirm that the length of G1 itself does not determine differentiation.

The question of whether there is a threshold level of PDCD2 protein required for its effects is interesting. In our studies, we found no evidence for altered growth for PDCD2 heterozygous ESCs, as well as for two of three MEF cell lines. In fact, in contrast to heterozygous MEFs, the heterozygous ESCs appeared to maintain approximately wild-type protein levels, suggesting the possible existence of mechanisms to maintain high levels of expression in these cells even in the presence of reduced gene dosage. The existence of such a mechanism would be interesting in view of the lack of effect of significant knockdown of PDCD2 protein levels in ESCs previously reported (Mu et al., 2010). However, growth inhibition was seen in the presence of small amounts of residual PDCD2 protein both in our Tam-treated ESCs, as well as in siRNA-mediated PDCD2 knockdown (but not complete knockout) in cancer cell lines (Barboza et al., 2013). Conversely, increased levels of PDCD2 expression have been shown to be associated with apoptosis following transfection of some transformed human cell lines (Baron et al., 2010). There are a number of possible explanations for the differences in PDCD2 “dosage” effects in different cell types. Clearly, cell type-specific regulation most likely contributes in one form or another. For example, knockdown of PDCD2 in ESCs resulted in a relative increase of nuclear versus cytoplasmic PDCD2 protein, potentially compensating for adverse effects due to decreased overall PDCD2 levels (Mu et al., 2010). Alternatively, it is possible that the unique cell cycle of ESCs allows for even small amounts of PDCD2 to provide sufficient activity permitting cell cycle progression.

In summary, using a series of inducible PDCD2 knockout mice and cell lines, we have demonstrated an essential role for PDCD2 in the proliferation of MEFS, ESCs and early mouse embryos. Our studies provide the key novel observation that PDCD2 plays a critical role in S phase entry. Furthermore, loss of PDCD2 is associated with the induction of p53 and of a subset of p53 target genes known to induce cell cycle arrest and cell death.

## MATERIALS AND METHODS

### Knockout mouse generation

PDCD2-targeted ESCs were purchased from the European Union Conditional Mouse Mutagenesis (EUCOMM) program (clones A09 and B12) (Pettitt et al., 2009; Skarnes et al., 2011). Knockout mice were generated by the CINJ/Rutgers-RWJMS transgenic and knockout mouse core facility. Two chimeras were generated from Albino-B6/NCI blastocyst injection of the clone A09. Both of these transmitted to the germline and have been maintained on the C57BL6/J background. Embryonic lethality of the homozygous progeny was confirmed for both lines by intercrossing heterozygous together. One line was used for all subsequent experiments.

### Embryo collection

Mouse breeding, euthanasia, and the collection of embryos were carried after approval of Rutgers-RWJMS IACUC, following institutional policies. Mice were maintained under a 12-hour light cycle (artificial daylight time between 7 am and 7 pm), and gestation (0.0 d*pc*) was considered to have begun at midnight before the morning when a copulation plug was found. Post-implantation embryos were recovered in DMEM containing 10% fetal bovine serum and 25 mM HEPES buffer. Pre-implantation embryos were recovered in M2 medium.

### Immunofluorescence microscopy of ESCs and preimplantation embryos

Immunofluorescence microscopy of ESCs and preimplantation embryos was adapted from a previously described protocol (Granier et al., 2011). Rabbit anti-p53 (CM5 Leica) was used as primary antibody at 1:400 dilution; Alexa-488 donkey anti-rabbit antibody (Invitrogen) was used as secondary antibody at 1:400. Embryos and cells were incubated at room temperature 3 h with primary antibody, and 1 h with secondary antibody. Cortical actin was counterstained with Alexa-568 Phalloidin (Invitrogen, A22287, dilution 1:50) at room temperature for 1 h. Nuclei were counterstained with Hoechst 33342 (5 µg/ml) for 15 min at RT. Images were acquired using a Zeiss LSM 700 confocal microscope (60× objective), then analyzed with ImageJ software. Paired *t* tests were used to determine whether the percentage of p53 positive nuclei per colonies and embryos were significantly different in knockout and controls.

### Preparation of inducible knockout MEFs

Primary mouse embryonic fibroblasts were prepared from 13.5 d*pc* embryos derived from breeding *PDCD2^flox/+^*, *R26-Cre-ER^T2^* mice with *PDCD2^lacZ/+^* mice (*R26-Cre-ER^T2^* mice, Jackson Laboratory, stock number 008463). Embryos were isolated from the uterine horn, the head and internal organs removed, and tail DNA isolated for genotyping. Each embryo was minced, treated with trypsin (0.25%, 15 min, 37°C), and grown in 10 cm plates in MEF medium (DMEM high glucose, 10% FBS, antibiotics, antimycotics and Non-essential amino acids). MEFs derived in this way were frozen at passages 1 and 2. For genotyping, the tails were lysed in Sigma Red-extract lysis buffer according to the manufacturer. 2 µl of the lysate was used for PCR amplification in a total volume of 10 µl using a mixture of LAR3, 3′arm and 5′arm primers (supplementary material Table S7). The PCR amplification protocol consisted of an initial incubation at 94°C for 3 min followed by 35 cycles of: 94°C for 30 s, 60°C for 30 s, and 72°C for 30 s, with a final incubation at 72°C for 10 min. Embryos were also genotyped for the presence of the *R26-Cre-ER^T2^* transgene using primers specific for Cre (Jackson Laboratory primers IMR1084 and IMR1085) with PCR amplification: 94°C for 3 min, 35 cycles at 94°C for 30 s, 51.7°C for 60 s, and 72°C for 30 s, and 72°C for 10 min. For each experiment, the induction of Cre, with resulting knockout of PDCD2 exon 2, was performed by incubating the cells with 1 µM Tam for 24 h.

### Preparation of inducible knockout mouse ESCs

ESCs were prepared from 3.5 d*pc* embryos derived from breeding *PDCD2^flox/+^*, *R26-Cre-ER^T2^* with *PDCD2^lacZ/+^* mice, using a previously described protocol (Bryja et al., 2006). Frozen stocks were prepared at passages 3 and 4, and cells were maintained on a feeder cell layer comprising Mitomycin C-arrested MEFs in a medium composed of Knockout DMEM supplemented with 20% ESC tested FBS, penicillin (100 U/ml)/Streptomycin (100 µg/ml), 2 mM l-glutamine, 1× minimal essential medium non-essential amino acids, 100 µm β-mercaptoethanol, and recombinant mouse leukemia inhibitory factor (1000 U/ml of ESGRO, Millipore). For each experiment, the induction of Cre with resulting knockout of PDCD2 exon 2 was performed by incubating the cells with 1 µM of Tam for 24 h.

### Growth curve analysis

Passage 1 *Pdcd2^+/+^*, *Pdcd2^+/lacZ^*, and *Pdcd2^lacZ/flox^* MEFs harboring the *R26-Cre-ER^T2^* transgene were plated at low density (5×10^4^ cells per well in 6 well plate), with or without 1 µM Tam. Medium was replaced by fresh medium without Tam after 24 h. Viable cells were counted by trypan blue staining in triplicate wells every 24 h for 6 days, starting 24 h after plating. The experiment was repeated twice with similar results. Growth of *Pdcd2^+/+^* and *Pdcd2^lacZ/flox^*, *R26-Cre-ER^T2^* transgene ESCs was monitored using the same protocol after initially plating ESCs at 3×10^5^ cells per well in 6 well plates.

### Cell cycle analyses of ESCs and MEFs

For DNA content analysis, 10^6^ cells were fixed in 1 ml of cold absolute ethanol. Cells were stored at −20°C at least 24 h, until the day of the staining. Cells were then washed twice with 3 ml of cold PBS and stained in 500 µl of PI/Triton X-100 staining solution (200 µg/ml of DNAse free RNase, 20 µg/ml of Propidium Iodide) at 4°C overnight. The cell suspension was filtered, and staining was analyzed at low flow rate by flow cytometry with FACSCalibur (BD Biosciences). At least 10,000 single cell events were acquired. FlowJo software (version 8.8.2, Watson algorithm) was used to estimate the fraction of cells in G_1_, S, and G_2_/M.

For measurement of proliferation by Click-iT analysis, MEFs (lines WT 3 and KO 3) were incubated for 1 h in 10 µM EdU, and labeled as previously described (Evertts et al., 2013). Briefly, 1 to 2×10^5^ cells were pelleted, fixed with 4% paraformaldehyde, permeabilized and treated with Alexa 488 azide (Invitrogen). DNA was stained with 4′, 6-diamidino-2-phenylindole (DAPI). Cells were analyzed with a LSRII flow cytometer (BD Biosciences). 100,000 events were analyzed per sample. FlowJo software (version 8.8.2) was used to analyze the fraction of cells in G_1_, S, and G_2_/M.

### RNA extraction, semi-qPCRs and real time PCRs analyses

RNA was extracted from MEFs and ESCs with TRIzol (Invitrogen) according to the manufacturer's protocol. cDNA was prepared from 500 ng of RNA using the Superscript VILO cDNA Syntheis Kit. 1 µl of 10-fold-diluted cDNA was then used for semi-qPCR or real time PCR. For semi-qPCR, the reaction was performed in 12.5 µl final volume (Qiagen Taq core kit), with 0.5 µM primers pairs for PDCD2 (Exon1F and Exon3R, Exon2F and Exon4R), and for GAPDH, (GAPDH-F and GAPDH-R), using a 62°C annealing temperature, and 30 seconds of elongation. The sequences of primers and predicted product sizes are shown in supplementary material Table S7. For real time quantitative PCR, reactions were performed in triplicate in 10 µl final volume, using the EXPRESS SYBR GreenER qPCR Supermix Universal (Invitrogen) at 60°C annealing temperature, employing the Mx3000P Stratagene qPCR machine. Primers used for real time qPCR are shown in supplementary material Table S5.

### Western blot analysis

For protein extraction, cells were washed twice with PBS, scraped and lysed in 1 ml of lysis buffer [50 mM of Tris pH 7.5, 0.5 M NaCl, 1% NP-40, 1% DOC, 0.1% SDS, 2 mM EDTA, 1× Complete protease inhibitor (Roche), phosphatase inhibitor cocktail (Roche)] per 10^7^ cells for 15–20 min on ice, then samples were sheared by passage through a 21 G hypodermic needle. Cellular debris was eliminated by centrifugation and lysate was stored for Western blot. Western blot was performed according to standard procedures using ECL detection (GE Healthcare). The following primary antibodies were used: rabbit anti-PDCD2 antibody at 1:2000 dilution (a kind gift from P. Sharp (Scarr and Sharp, 2002)), rabbit anti-p53 antibody at 1:2500 (CM5, Leica), rabbit anti-PERP antibody at 1:1000 (Abcam), rabbit anti-p21antibody at 1:1000 (Abcam), and mouse anti-β-actin antibody at 1:5000 (AC-15, Sigma). Horseradish peroxidase-conjugated anti-rabbit (Santa-Cruz), or anti-mouse antibodies at a 1:5000 dilution (Cell Signaling Technology) were used as secondary antibodies. Quantification of protein levels was measured using Image Studio software.

### Cell cycle analysis of mouse embryos

For measurement of proliferation by Click-iT analysis, 3.5 d*p*c embryos were harvested in M2 medium and cultured for 1 h in M16 medium supplemented with 10 µM EdU (Invitrogen), then labeled according to the manufacturer's protocol (Click-iT EdU Imaging kit, alexa fluor 488 Azide). Phospho-Histone H3 (Ser10) labeling was performed as described above using mouse anti-Phospho-Histone H3 (Ser10) antibody at 1:1000 dilution (Sigma), followed by anti-mouse Alexa 568 (Invitrogen) at 1:400. Embryos were then counter-stained with Hoechst 33342 for 15 min at RT, and images acquired by confocal microscopy (Zeiss LSM 700). ImageJ software was used to analyze the percentage of cells in G_1_, S, and G_2_/M. Paired *t* tests were used to determine whether the percentage of cells in early S phase and G1 phase were significantly different in knockout embryos and controls.

### Genotyping pre-implantation embryos and outgrowths

After microscopic analysis, samples were individually lysed in 12.5 µl of Sigma Red-extract lysis buffer. 2 µl of the lysate was used for a first round of PCR in a total volume of 10 µl. The PCR amplification protocol consisted of an initial incubation at 94°C for 3 min followed by 35 cycles at 94°C for 30 s, 60°C for 30 s, and 72°C for 30 s, with a final incubation at 72°C for 10 min. A total of 4 µl of the first PCR was used for a second round of PCR amplification for 20 cycles. For embryos derived from *Pdcd2^+/−^* and *Pdcd2^+/lacZ^* intercrosses, the first round of PCR used a mixture of LAR3, 3′arm and 5′arm primers and the second round of PCR used a mixture of Nested LAR3, Nested 3′arm and Nested 5′arm primers (supplementary material Table S7). For embryos derived from *Pdcd2^Δexon2^* intercrosses, first round PCR used a mixture of Exon3-R, 3′arm and 5′arm primers, and the second round of PCR used a mixture of Nested-Exon3-R, Nested 3′arm and Nested 5′arm primers (supplementary material Table S7).

### RNA-seq on inducible knockout MEFs

Total RNA was prepared from MEF lines WT 2 and 3, and KO 2 and 3, and was subjected to either enrichment for polyA containing RNA (TruSeq RNA Sample preparation V2 kit, Illumna) or rRNA depletion (RiboMinus human/mouse module, Invitrogen). These enriched RNA samples were converted into RNA-seq libraries with different DNA barcodes using the Illumina TruSeq RNA Sample Prep Kits V2. The libraries were examined on Bioanalyzer (Agilent) HS chips for size distribution, and quantified by Qubit fluorometer (Invitrogen). Each set of 4 libraries was pooled together and sequenced on Illumina HiSeq 2000 as one lane of single-end, 101 nt reads, following the standard protocol. Duplicate sets of 4 libraries were prepared and sequenced. Raw sequencing reads were filtered by Illumina HiSeq Control Software to generate Pass-Filter (PF) reads for further analysis.

PF Reads were de-multiplexed using the Barcode Splitter in FASTX-toolkit and the reads from each library were mapped to mouse genome build mm10 with RefSeq gene annotation, using Tophat 2 software, allowing only unique alignment for each read. The htseq-count software was used next to obtain gene expression value as the number of reads mapped to all exons of each gene, and these counts were further normalized to counts on each gene per 10 million mapped reads in each sample. The normalized gene expression values of each sample were log2-transformed, after raising all zero values to 0.5, to obtain log2-fold change between each pair of Flox-Tam and WT-Tam samples. Differential genes were selected in each group of samples (polyA^+^ libraries only, Ribo^−^ libraries only, or all 8 samples combined) as having mean fold change over 1.5-fold, and mean expression value over 16 among all samples in the group.

## Supplementary Material

Supplementary Material
