# Supplementary material for: Conditional inactivation of PDCD2 induces p53 activation and cell cycle arrest
Source: Biol Open. 2014 Aug 22;3(9):821–31. doi: 10.1242/bio.20148326 (PMC4163659; doi:10.1242/bio.20148326)
Supplement: Supplementary Material [file supp_3_9_821__index.html]

Conditional inactivation of PDCD2 induces p53 activation and cell cycle arrest — Supplementary Material 

# Conditional inactivation of PDCD2 induces p53 activation and cell cycle arrest

## bio.20148326 Supplementary Material

**Files in this Data Supplement:**

- Supplementary Material - Celine J. Granier et al. doi: 10.1242/bio.20148326
- Table S1 - Genotype analysis of *Pdcd2+/−* intercross progeny
- Table S2 - Genotype analysis of *Pdcd2+/Δexon2* intercross progeny
- Table S3 - RNA-seq of polyadenylated RNAs in Tam-treated *Pdcd2Flox/lacZ* and WT MEFs. RNA-seq data for the 901 genes from the polyA+ RNA (denoted as "a") samples exhibiting a mean expression value (read number) over 16, and a mean fold change greater than or equal to 1.5-fold comparing differentially expressed genes between *Pdcd2Flox/lacZ* + Tam (called FxTm-1 and FxTm-2) and *WT* + Tam called (WtTm-1 and -2) in two replicate RNA-seq experiments (experiments 1 and 2).
- Table S4 - RNA-seq of Ribo− RNAs in Tam-treated *Pdcd2Flox/lacZ* and WT MEFs. RNA-seq data for the 886 genes from the ribosomal RNA-depleted (denoted as "r") samples exhibiting a mean expression value (read number) over 16, and a mean fold change greater than or equal to 1.5-fold in samples when comparing differentially expressed genes between *Pdcd2Flox/lacZ* + Tam (called FxTm-1 and -2), *WT* + Tam called (WtTm-1 and -2) in two replicate RNA-seq experiments (experiments 1 and 2).
- Table S5 - RNA-seq of RNAs exhibiting >1.5-fold change in Tam-treated *Pdcd2Flox/lacZ* versus WT MEFs in both polyA+ and Ribo− RNA samples. RNA-seq data for the 509 genes exhibiting a mean expression value (read number) over 16, and a mean fold change greater than or equal to 1.5-fold in samples when comparing differentially expressed genes between *Pdcd2Flox/lacZ* + Tam (called FxTm-1 and -2), *WT* + Tam called (WtTm-1 and -2) in across both polyA+ and Ribo− duplicate RNA-seq experiments (experiments 1 and 2).
- Table S6 - E2F target genes downregulated >2-fold in MEFs by PDCD2 knockout
- Table S7 - Lists of primers
