# Supplementary material for: Conditional inactivation of PDCD2 induces p53 activation and cell cycle arrest
Source: Biol Open. 2014 Aug 22;3(9):821–31. doi: 10.1242/bio.20148326 (PMC4163659; doi:10.1242/bio.20148326)
Supplement: Supplementary Material [file supp_bio.20148326_bio.20148326-s1.pdf]

Biology Open (2014) 000, 1–11 doi:10.1242/bio.20148326

Supplementary Material

Celine J. Granier et al. doi: 10.1242/bio.20148326

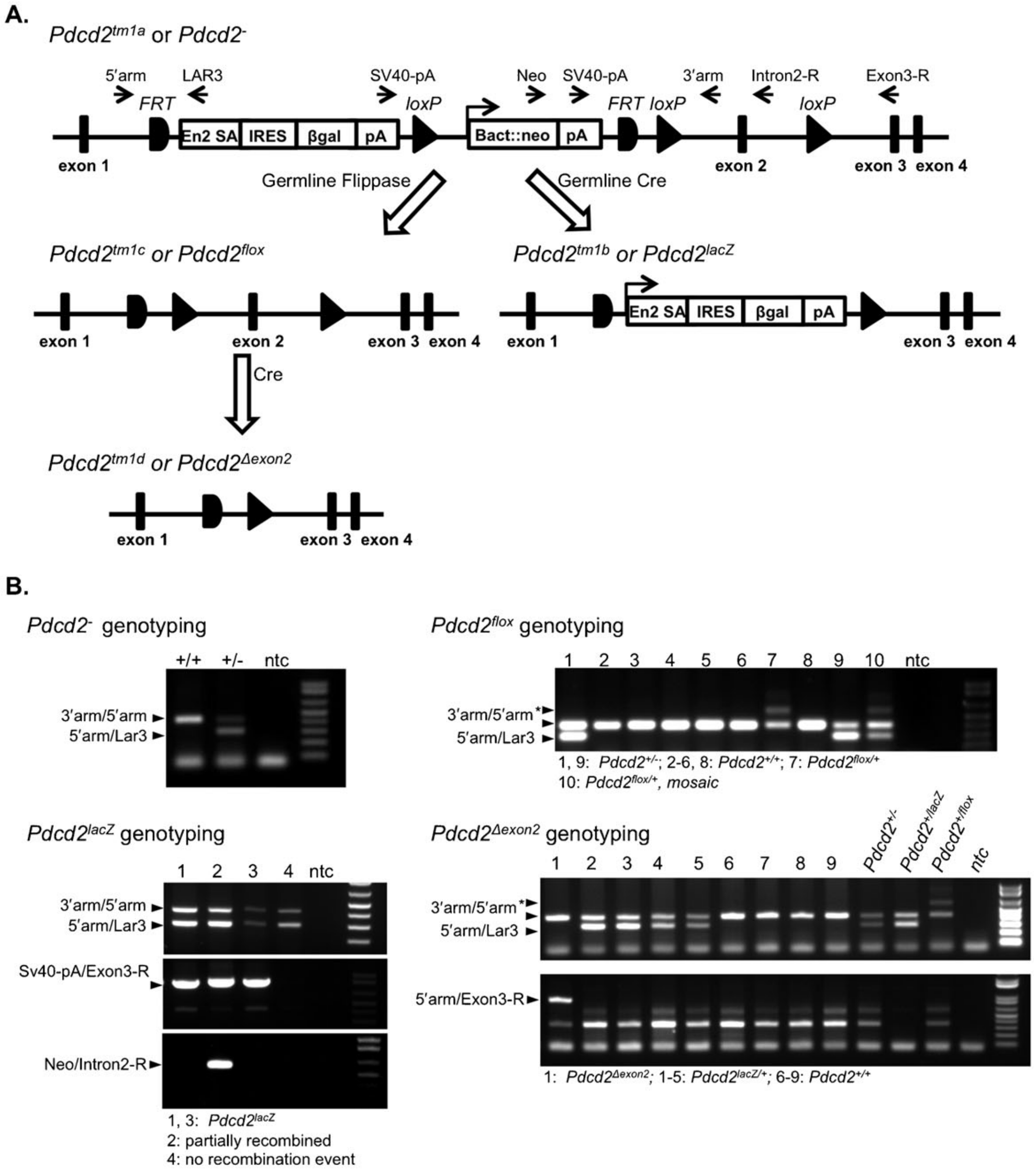

Fig. S1. Generation of different *Pdcd2* knockout alleles. (A) Strategy for generation of *Pdcd2* knockout mice. The original targeted ''knockout-first'' allele from EUCOMM ($Pdcd2^{tm1a}$ or $Pdcd2^{-}$) contains a targeting cassette that includes the mouse En2 splice acceptor and the SV40 polyadenylation sequences, which are predicted to generate a null allele through splicing to a lacZ ''gene trap'' element. *ActB-Flpe* transgenic mice (expressing the Flippase in the germline) were crossed to $Pdcd2^{+/-}$ mice in order to generate a conditional allele ($Pdcd2^{tm1c}$ or $Pdcd^{flox}$) for deletion of exon2, which is flanked by *lox p* sites. *Sox2-Cre* female mice (expressing Cre in the germline) were crossed to $Pdcd2^{+/-}$ male mice in order to remove the *neo* selection cassette and exon2, generating a *lacZ*-tagged allele ($Pdcd2^{tm1b}$ or $Pdcd2^{lacZ}$), also predicted to function as a ''gene trap'' null allele through splicing to the *lacZ* trapping element. $Pdcd2^{+/flox}$ male mice were crossed to $Pdcd2^{+/lacZ}$; *Sox2-Cre* females in order to generate a knockout allele deleted for exon2 ($Pdcd2^{tm1d}$ or $Pdcd2^{\Delta exon2}$). (B) PCRs strategies for genotyping *Pdcd2* knockout mice. Genotyping of $Pdcd2^{-}$, $Pdcd2^{flox}$, $Pdcd2^{lacZ}$ and $Pdcd2^{\Delta exon2}$ was carried out by triplex PCR using primers: DNA PCR using 5′arm, 3′arm, and Lar3. The 5′arm/Lar3 band is only present in the $Pdcd2^{-}$ and $Pdcd2^{lacZ}$ alleles. For the $Pdcd2^{flox}$ genotyping, the asterisk shows a third PCR product with 3′arm and 5′arm primers that is amplified if the flippase recombination event occurred. For $Pdcd2^{lacZ}$, genotyping was also carried out with primers Sv40-pA and Exon3-R to identify the exon2 deletion event, and primers Neo and Intron2-R for the presence or absence of the neo cassette. For $Pdcd2^{\Delta exon2}$ genotyping was also carried out by PCR using primers 5′arm and Exon3-R for the exon2 deletion event. The asterisk shows a second PCR product obtained with the 3′arm and 5′arm that is amplified if the Cre-mediated recombination of the flox allele fails to occur. Sizes expected of PCR products for each primer pair and each template are listed in supplementary material Table S7. ntc indicates the no template control.

Biology Open (2014) 000, 1–11 doi:10.1242/bio.20148326

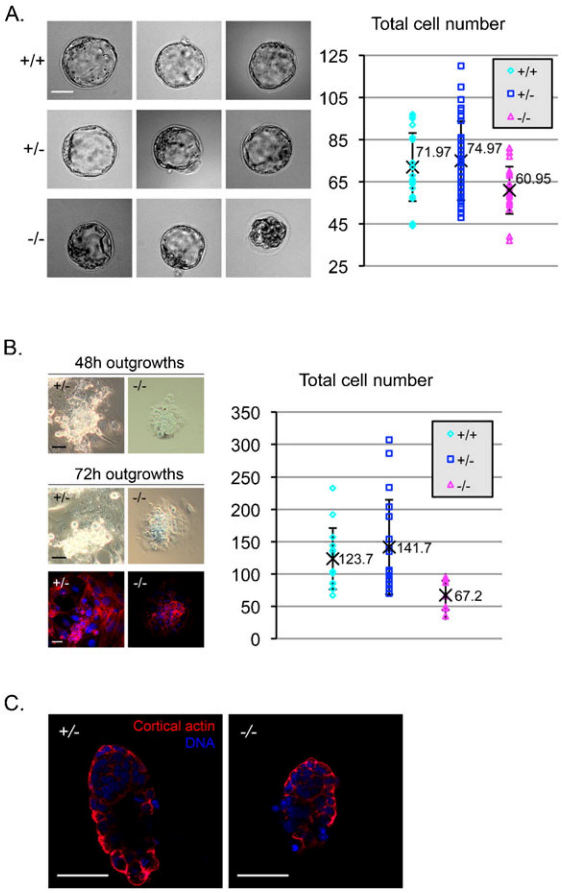

Fig. S2. PDCD2 knockout results in loss of embryonic growth. (A) Analysis of 3.5 d*pc* embryos, size and morphology. Left panel: 3.5 d*pc* *Pdcd2*$^{-/-}$ embryos are smaller than WT embryos but do not show other drastic morphologic changes. Right panel: total cell numbers (TCN) (i.e. Hoechst positive cells) of 3.5 d*pc* embryos were quantitated. *Pdcd2*$^{-/-}$ embryos have fewer cells than the majority of littermate control embryos. Mutant embryo cell numbers are in the lower range of TCN compared to their littermate control (+/+ avg TCN=72, N=20; +/− avg. TCN=75, N=36; −/− avg TCN=61, N=22; *t* test shows no significant difference between +/+ and +/− (p=0.58), and significant difference between −/− and +/− (p=0.0025) and between −/− and +/+ (p=0.01)). Scale bar: 50 µm. (B) Analysis of *ex vivo* embryo outgrowths. 3.5 d*pc* blastocysts were placed for 24 h to 72 h in ESC medium, on 0.1% gelatin-coated chambered coverglass. Left panel: brightfield and confocal images (blue = DNA; red = cortical actin) of 48 h and 72 h outgrowths showing loss of growth of *Pdcd2*$^{-/-}$ compare to the littermate controls. Right panel: total cell number (Hoechst positive cells) of 72 h outgrowths. *Pdcd2*$^{-/-}$ outgrowths show a significantly lower TCN (average 67.2, N=6) compared to their littermate control (+/+, average 123.7, N=11; +/−, average 141.7, N=17), (p=0.0004, *t* test). (C) Confocal section of representative 4.5 d*pc* embryos labeled with Hoechst (blue) and Phalloidin showing cortical actin (red). At this stage, the size of the knockout embryos is smaller than their littermate controls. Scale bars: 50 µm.

Biology Open (2014) 000, 1–11 doi:10.1242/bio.20148326

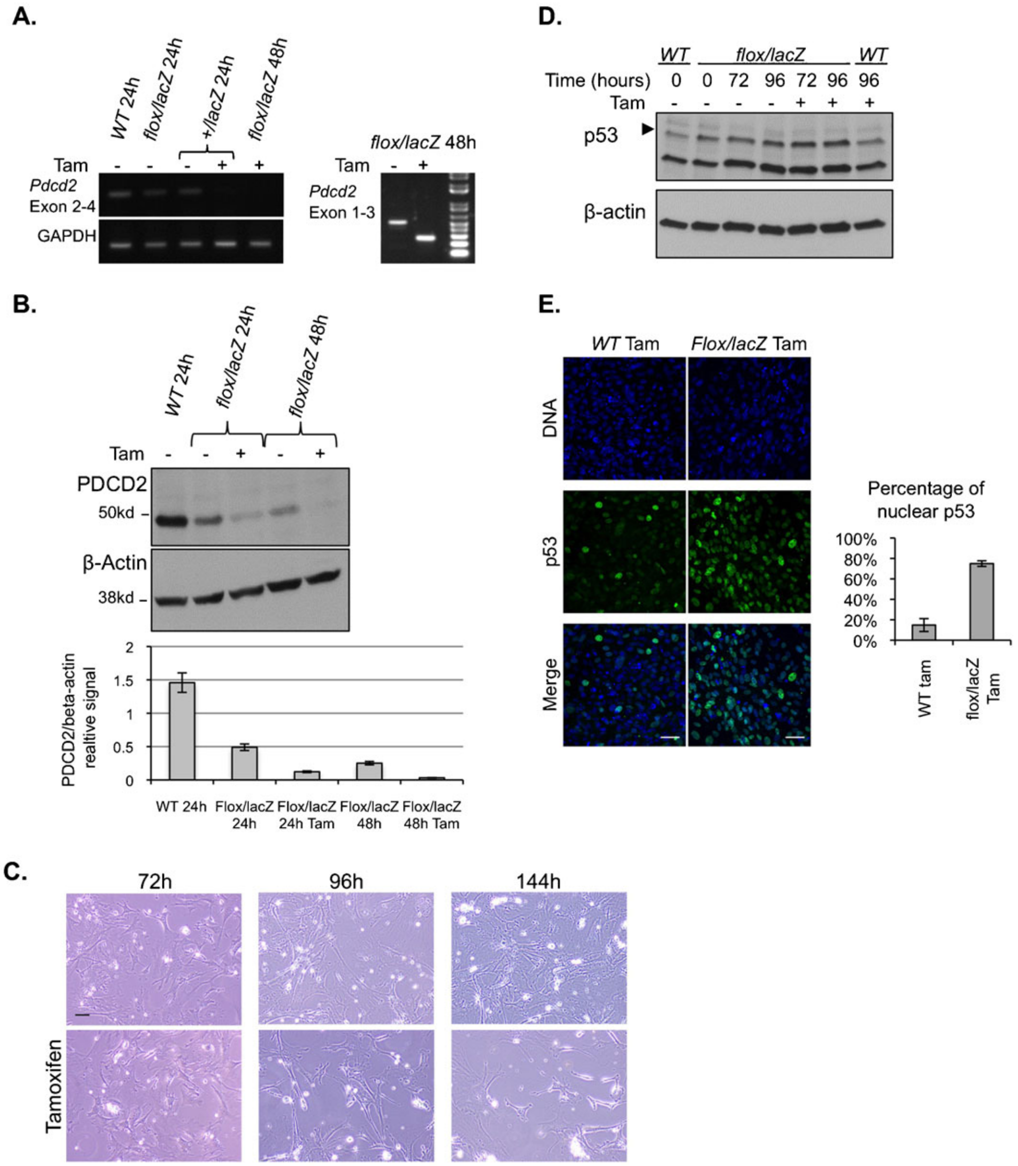

Fig. S3. Characterization of PDCD2 inducible knockout in MEFs. (A) Left panel: PCR of reverse transcribed PCR of *Pdcd2* RNA compared to GAPDH RNA, showing loss of PDCD2 expression at 24 h. Right panel: PCR of *Pdcd2* RNA from *Pdcd2*$^{flox/lacZ}$ showing the presence of a truncated transcript in presence of Tam, seen with amplification using primers from exons 1 and 3. Primers and expected sizes of amplified products are listed in supplementary material Table S5. (B) Western blot analysis for PDCD2, showing lower amount of PDCD2 protein in *Pdcd2*$^{flox/lacZ}$ compared to *Pdcd2*$^{+/+}$ MEFs in absence of Tam, and a complete loss of PDCD2 protein 48 h after Tam treatment. (C) Morphological analysis of *Pdcd2*$^{flox/lacZ}$ inducible knockout MEFs, in presence or absence of Tam. Scale bar: 10 μm. (D) Western blot analysis of p53 protein levels in *WT* and *Pdcd2*$^{flox/lacZ}$ cells with and without Tam treatment. β-actin protein levels are shown as a loading control. (E) Immunofluorescence for p53 protein 96 h after Tam treatment of WT and *Pdcd2*$^{flox/lacZ}$ MEFs. Left panel: confocal acquisitions of MEFs stained for DNA (Hoechst, blue) and p53 (green); right panel: quantification of percentage of cells with nuclear p53 cells (TCN quantified based on Hoechst). More cells with nuclear p53 are present in *Pdcd2*$^{flox/lacZ}$ MEFs (average 74.97%, N=230) than WT ESCs (average 14.82%, N=359), 96 h after Tam treatment. Scale bars: 50 μm.

Biology Open

Biology Open (2014) 000, 1–11 doi:10.1242/bio.20148326

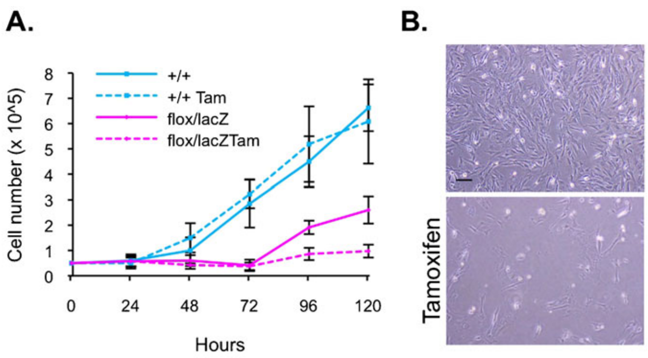

Fig. S4. Inducible knockout of PDCD2 in immortalized MEFs results in loss of cell growth. MEFs (Lines WT 1 and KO 1) from passage one (P1) was plated at a density of 1.5×10^4 cells per cm^2 and passaged every third day. This re-plating procedure was repeated for 20 passages (P20) to achieve immortalized cells (Xu, 2005). (A) Growth curve of inducible knockout and control immortalized MEFs, in presence or absence of tamoxifen (TAM). *Pdcd2*$^{flox/lacZ}$ MEFs exhibit slowed growth compared to *WT* MEFs, even in the absence of Tam. Tam-treated *Pdcd2*$^{flox/lacZ}$ MEFs exhibit a cessation of growth following Tam treatment. (B) Morphological analysis of *Pdcd2*$^{flox/lacZ}$ inducible knockout MEFs in presence or absence of Tam. Scale bar: 10 µm.

Biology Open

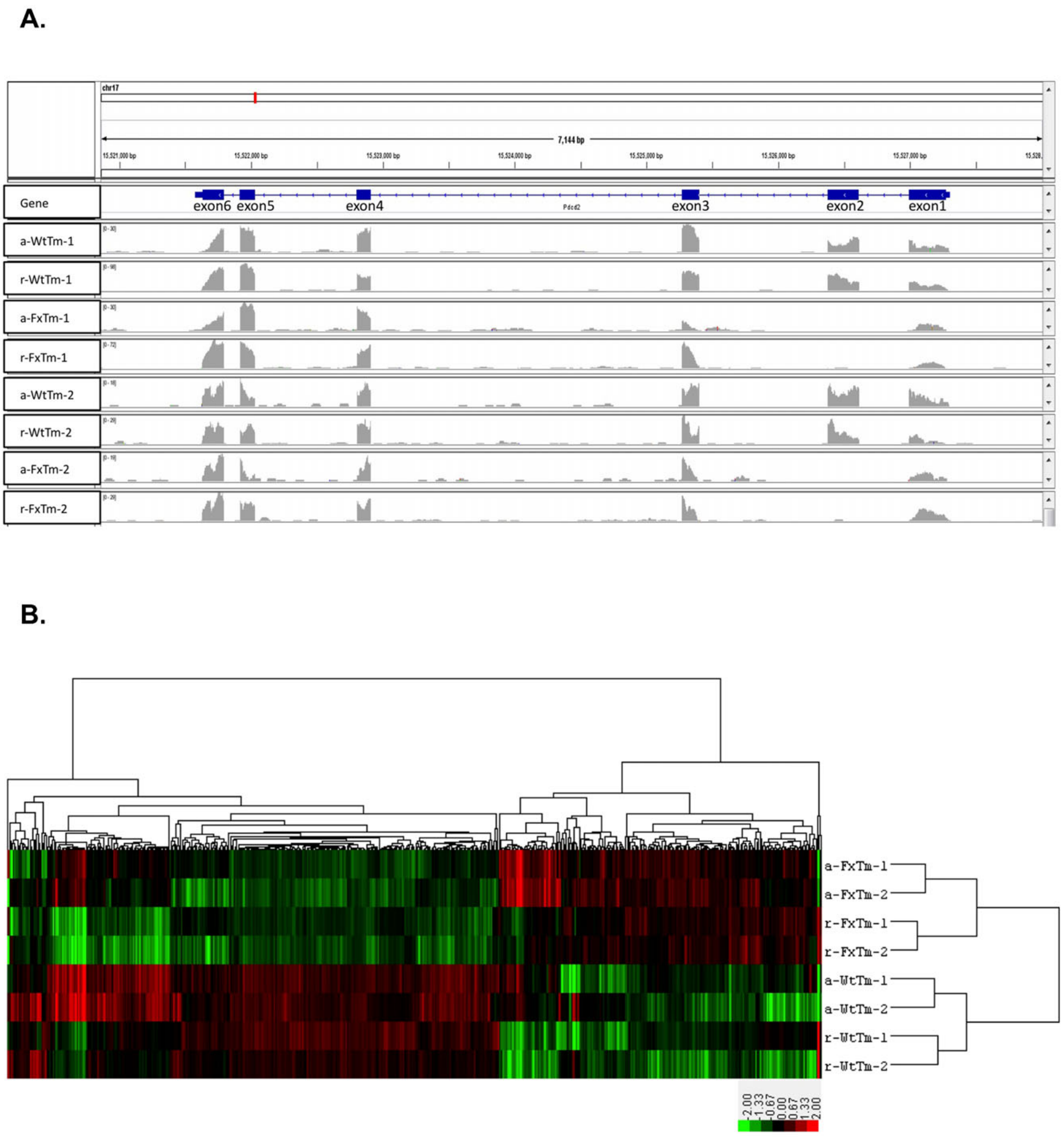

Fig. S5. RNA seq analysis on inducible knockout MEFs. (A) Read alignment coverage of the *Pdcd2* gene in all 8 polyA$^+$ and Ribo$^-$ RNA libraries. The read coverage of exon2 was zero in the *Pdcd2$^{Flox/lacZ}$* (FxTm) polyA$^+$ samples and essentially zero in the Ribo$^-$ samples (only 2–3 mapped reads, comparable to the intron coverage shown here). (B) Hierarchical clustering analysis of all 8 polyA$^+$ RNA samples and Ribo$^-$ RNA samples using normalized gene expression values of the top 509 genes differentially expressed between Tam-treated *Pdcd2$^{Flox/lacZ}$* and Tam-treated WT cells. Within each set of four polyA$^+$ (called a) samples or Ribo$^-$ (called r) samples (two samples from *Pdcd2$^{Flox/lacZ}$* + Tam called FxTm-1 and -2, and two samples from *WT* + Tam called WtTm-1 and -2), differentially expressed genes were selected on the basis of a mean expression value (read number) over 16, and a mean fold change greater than or equal to 1.5-fold across all 4 samples. This yielded 901 genes from polyA$^+$ samples and 886 genes from Ribo$^-$ samples, of which 509 genes overlapped. The expression values of these 509 genes were log2-transformed and centered by subtracting the gene-wise medians. The genes (in columns) and samples (in rows) were clustered using the Pearson correlation coefficient and average linkage algorithm. Overall, these samples cluster by genotype, then by the two RNA enrichment methods.

Biology Open (2014) 000, 1–11 doi:10.1242/bio.20148326

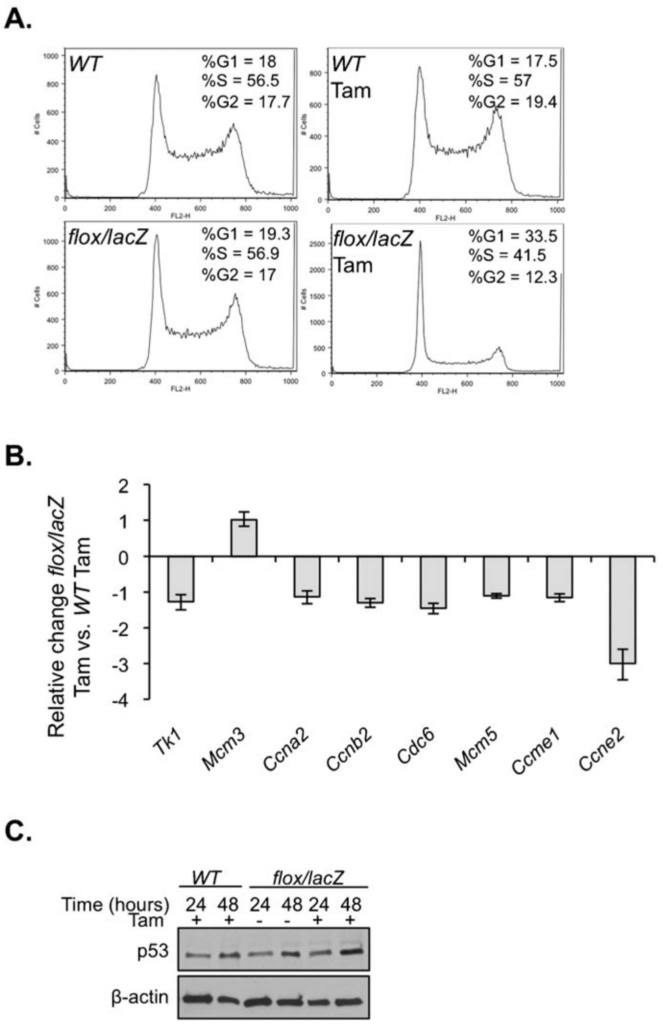

Fig. S6. PDCD2 knockout in ESCs induces morphological changes, loss of S phase entry, and increased p53 levels. (A) Cell cycle analysis 120 h after Tam treatment of *Pdcd2*$^{+/+}$ and *Pdcd2*$^{flox/lacZ}$ ESCs. (B) qPCR analysis of E2F target genes RNA levels. Results represent the fold change of *Pdcd2*$^{flox/lacZ}$ compared to *Pdcd2*$^{+/+}$ cells 96 h after Tam treatment. (C) Western blot analysis of p53 protein levels in *WT* and *Pdcd2*$^{flox/lacZ}$ cells at 24 h and 48 h after initial tamoxifen treatment. β-actin protein levels are shown as a loading control.

Biology Open
