# Supplementary material for: Conditional inactivation of PDCD2 induces p53 activation and cell cycle arrest
Source: Biol Open. 2014 Aug 22;3(9):821–31. doi: 10.1242/bio.20148326 (PMC4163659; doi:10.1242/bio.20148326)
Supplement: Supplementary Material [file supp_bio.20148326_Table_S1.docx]

| **Table S1. Genotype analysis of *Pdcd2^+/-^* intercross progeny** | | | | |
| --- | --- | --- | --- | --- |
| Stage | Number by genotype | | | Total number |
|  | *Pdcd2*^+/+^ | *Pdcd2^+/-^* | *Pdcd2^-/-^* |  |
| Weaning age*^a^* | 13 | 38 | 0 | 51 |
| 6.5d*pc^b^* | 9 | 17 | 0 | 26 |
| 4.5d*pc^c^* | 0 | 7 | 3 | 10 |
| 3.5d*pc^d^* | 29 | 40 | 27 | 96 |

*^a^*Number of progeny coming from 6 litters. *^b^*Number of progeny coming from 4 litters. *^c^*Number of progeny coming from 1 litter. *^d^*Number of progeny coming from 13 litters.
