# Supplementary material for: Conditional inactivation of PDCD2 induces p53 activation and cell cycle arrest
Source: Biol Open. 2014 Aug 22;3(9):821–31. doi: 10.1242/bio.20148326 (PMC4163659; doi:10.1242/bio.20148326)
Supplement: Supplementary Material [file supp_bio.20148326_Table_S2.docx]

| **Table S2: Genotype analysis of *Pdcd2^+/Δexon2^* intercross progeny** | | | | |
| --- | --- | --- | --- | --- |
| Stage | Number by genotype | | | Total number |
|  | *Pdcd2*^+/+^ | *Pdcd2^+/Δexon2^* | *Pdcd2^Δexon2/Δexon2^* |  |
| Weaning age*^a^* | 16 | 13 | 0 | 29 |
| 5.5d*pc^b^* | 10 | 10 | 0 | 20 |
| 4.5d*pc^c^* | 1 | 3 | 3 | 7 |
| 3.5d*pc^d^* | 5 | 5 | 3 | 13 |

*^a^*Number of progeny coming from 4 litters. *^b^*Number of progeny coming from 4 litters. *^c^*Number of progeny coming from 1 litter. *^d^*Number of progeny coming from 2 litters.
