# Supplementary material for: Conditional inactivation of PDCD2 induces p53 activation and cell cycle arrest
Source: Biol Open. 2014 Aug 22;3(9):821–31. doi: 10.1242/bio.20148326 (PMC4163659; doi:10.1242/bio.20148326)
Supplement: Supplementary Material [file supp_bio.20148326_Table_S6.docx]

**Table S6: E2F Target Genes Downregulated >2 fold in MEFs by PDCD2 Knockout**

| **Genes** | **Fold (log2)** |  | **Genes** | **Fold (log2)** |
| --- | --- | --- | --- | --- |
| Oip5 | **-1.51** |  | Bub1b | **-1.21** |
| Exo1 | **-1.51** |  | Fancb | **-1.20** |
| Cdc25c | **-1.50** |  | Ttk | **-1.20** |
| Cdc6 | **-1.43** |  | Cenpf | **-1.20** |
| Aspm | **-1.42** |  | Foxm1 | **-1.19** |
| Ccnb2 | **-1.42** |  | Mad2l1 | **-1.19** |
| Iqgap3 | **-1.40** |  | Kif20b | **-1.19** |
| Fancd2 | **-1.39** |  | Sgol1 | **-1.19** |
| Melk | **-1.39** |  | Ccne2 | **-1.18** |
| Mybl2 | **-1.38** |  | Brca1 | **-1.18** |
| Pbk | **-1.37** |  | Mcm10 | **-1.17** |
| Top2a | **-1.36** |  | Brip1 | **-1.17** |
| Kif11 | **-1.35** |  | Lig1 | **-1.16** |
| Aurkb | **-1.33** |  | Mcm8 | **-1.14** |
| Rad54l | **-1.32** |  | Cenpa | **-1.13** |
| Rad54b | **-1.30** |  | Racgap1 | **-1.12** |
| Cdca8 | **-1.29** |  | Mcm3 | **-1.11** |
| Cdca3 | **-1.28** |  | Cdc20 | **-1.10** |
| Asf1b | **-1.28** |  | Cdc45 | **-1.08** |
| Kif2c | **-1.28** |  | Rpa2 | **-1.07** |
| Fbxo5 | **-1.27** |  | Kif20a | **-1.06** |
| Ccna2 | **-1.27** |  | Ccnb1 | **-1.06** |
| Kntc1 | **-1.27** |  | Ung | **-1.06** |
| Kif4 | **-1.27** |  | Cenpe | **-1.04** |
| Tk1 | **-1.25** |  | Plk4 | **-1.04** |
| Mcm5 | **-1.25** |  | Fanca | **-1.03** |
| Rad51ap1 | **-1.24** |  | Tpx2 | **-1.03** |
| Kif15 | **-1.24** |  | Kif24 | **-1.02** |
| Bub1 | **-1.23** |  | Pola1 | **-1.01** |
| Chek1 | **-1.23** |  | Lmnb1 | **-1.01** |
| Plk1 | **-1.23** |  | Kif23 | **-1.00** |
| E2f2 | **-1.22** |  | Arhgap19 | **-1.00** |
| Hmgb2 | **-1.21** |  |  |  |

Red: E2F targets from reference (Bracken et al., 2004)

Green: Top E2F targets by ChIP, reference (Xu et al., 2007)

Blue: E2F targets from gProfiler analysis

Red: E2F targets observed in both references (Bracken et al., 2004; Xu et al., 2007)

Other: E2F targets from other gene-specific references (not shown)
