# Supplementary material for: Conditional inactivation of PDCD2 induces p53 activation and cell cycle arrest
Source: Biol Open. 2014 Aug 22;3(9):821–31. doi: 10.1242/bio.20148326 (PMC4163659; doi:10.1242/bio.20148326)
Supplement: Supplementary Material [file supp_bio.20148326_Table_S7.docx]

**Table S7. Lists of primers**

| **Primers for genotyping** | Forward (5’-3’) | Reverse (5’-3’) | Amplicon size (bp) |
| --- | --- | --- | --- |
| *Pdcd2^-^* and *Pdcd2^lacZ^* | 5’arm: GAACCCTGGACGTAGGACGATCGG | LAR 3: CAACGGGTTCTTCTGTTAGTCC | 309 |
|  | Nested 5’arm: ATCGGTTGGCGCGAGTGGTT | Nested LAR 3: GGGCAAGTGTGGAGGGTGGT | 232 |
| *Pdcd2^lacZ^*, excised exon2 | SV40-pA: ACCTCCCACACCTCCCCCTGA | Exon3-R: CCTCCATTTCCACAACCTCAGGCA | 894 |
| Partially excised *Pdcd2^lacZ^* | Neo: TATCGCCTTCTTGACGAGTTC | Intron2-R: TGAACTGATGGCGAGCTCAGACC | 595 |
| *Pdcd2^flox^* | 5’arm: GAACCCTGGACGTAGGACGATCGG | 3’arm: TGAGCTGGAGGCACACACATTCCG | 496 |
| *Pdcd2^Δexon2^* | 5’arm: GAACCCTGGACGTAGGACGATCGG | Exon3-R: CCTCCATTTCCACAACCTCAGGCA | 922 |
|  | Nested 5’arm: ATCGGTTGGCGCGAGTGGTT | Nested Exon3-R: GAACTGATGGCGAGCTCAGA | 260 |
| *Pdcd2^+^* | 5’arm: GAACCCTGGACGTAGGACGATCGG | 3’arm: TGAGCTGGAGGCACACACATTCCG | 428 |
|  | Nested 5’arm: ATCGGTTGGCGCGAGTGGTT | Nested 3’arm: GCTTTTAAACCCGGGAAGAG | 128 |

| **Primers for semi-qPCR** | Forward (5’-3’) | Reverse (5’-3’) | Amplicon size (bp) |
| --- | --- | --- | --- |
| *Pdcd2* | Exon1F: TGTGGAGTTGGGCTTCGCCG | Exon3R: cctccatttccacaacctcaggca | 605 |
| *Pdcd2* after cre excision on *Pdcd2^flox^* | Exon1F: TGTGGAGTTGGGCTTCGCCG | Exon3R: cctccatttccacaacctcaggca | 365 |
| *Pdcd2* | Exon2F: CAGACATTAGACTGGCGGCT | Exon4R: TCTTCCTTGGATTCGTGCTT | 236 |
| *GAPDH* | GAPDH-F: TCTGCCGATGCCCCCATGTT | GAPDH-R: CCACAGCCTTGGCAGCACCA | 280 |

| **Primers for real-time PCR** | Forward (5’-3’) | Reverse (5’-3’) |
| --- | --- | --- |
| *GAPDH* | TGTAGACCATGTAGTTGAGGTCA | AGGTCGGTGTGAACGGATTTG |
| *Tk1* | AAGTGCCTGGTCATCAAGTATG | GCTGCCACAATTACTGTCTTGC |
| *Ccnb2* | GCCAAGAGCCATGTGACTATC | CAGAGCTGGTACTTTGGTGTTC |
| *Mcm3* | AGCGCAGAGAGACTACTTGGA | GCGGTTAGCCCTCTTTTCATTC |
| *Ccne1* | GAAAAGCGAGGATAGCAGTCAG | CCCAATTCAAGACGGGAAGTG |
| *Ccna2* | GCCTTCACCATTCATGTGGAT | TTGCTCCGGGTAAAGAGACAG |
| *Mcm5* | GGGCATTTTCTACAGCGACAG | GAACTCCTTGAATCGCCTCTG |
| *Ccne2* | GCTGATTCCTCCAGACAGTACA | ATGTCAAGACGCAGCCGTTTA |
| *Cdc6* | TGGCATCATACAAGTTTGTGTGG | CAGGCTGGACGTTTCTAAGTTTT |
| *Oct4* | CGGAAGAGAAAGCGAACTAGC | ATTGGCGATGTGAGTGATCTG |
| *Nanog* | TCTTCCTGGTCCCCACAGTTT | GCAAGAATAGTTCTCGGGATGAA |
| *Klf4* | GTGCCCCGACTAACCGTTG | GTCGTTGAACTCCTCGGTCT |
| *Sox2* | GCGGAGTGGAAACTTTTGTCC | CGGGAAGCGTGTACTTATCCTT |
| *Mdm2* | TGTCTGTGTCTACCGAGGGTG | TCCAACGGACTTTAACAACTTCA |
| *Perp* | ATCGCCTTCGACATCATCGC | CCCCATGCGTACTCCATGAG |
| *Exo1* | TGGCTGTGGATACCTACTGTT | ATCGGCTTGACCCCATAAGAC |
| *Cdc25c* | ATGTCTACAGGACCTATCCCAC | ACCTAAAACTGGGTGCTGAAAC |
| *P21 (CDKN1A)* | CCT GGT GAT GTC CGA CCT G | CCA TGA GCG CAT CGC AAT C |
